# Simple-to-use CRISPR-SpCas9/SaCas9/AsCas12a vector series for genome editing in *Saccharomyces cerevisiae*

**DOI:** 10.1093/g3journal/jkab304

**Published:** 2021-08-30

**Authors:** Satoshi Okada, Goro Doi, Shitomi Nakagawa, Emiko Kusumoto, Takashi Ito

**Affiliations:** Department of Biochemistry, Kyushu University Graduate School of Medical Sciences, Fukuoka 812-8582, Japan

**Keywords:** genome editing, CRISPR/Cas, *Saccharomyces cerevisiae*, budding yeast, SpCas9, SaCas9, AsCas12a, Golden Gate Assembly

## Abstract

Genome editing using the CRISPR/Cas system has been implemented for various organisms and becomes increasingly popular even in the genetically tractable budding yeast *Saccharomyces cerevisiae*. Because each CRISPR/Cas system recognizes only the sequences flanked by its unique protospacer adjacent motif (PAM), a certain single system often fails to target a region of interest due to the lack of PAM, thus necessitating the use of another system with a different PAM. Three CRISPR/Cas systems with distinct PAMs, namely SpCas9, SaCas9, and AsCas12a, have been successfully used in yeast genome editing. Their combined use should expand the repertoire of editable targets. However, currently available plasmids for these systems were individually developed under different design principles, thus hampering their seamless use in the practice of genome editing. Here, we report a series of Golden Gate Assembly-compatible backbone vectors designed under a unified principle to exploit the three CRISPR/Cas systems in yeast genome editing. We also created a program to assist the design of genome-editing plasmids for individual target sequences using the backbone vectors. Genome editing with these plasmids demonstrated practically sufficient efficiency in the insertion of gene fragments to essential genes (median 52.1%), the complete deletion of an open reading frame (median 78.9%), and the introduction of single amino acid substitutions (median 79.2%). The backbone vectors with the program would provide a versatile toolbox to facilitate the seamless use of SpCas9, SaCas9, and AsCas12a in various types of genome manipulation, especially those that are difficult to perform with conventional techniques in yeast genetics.

## Introduction

Clustered Regularly Interspaced Short Palindromic Repeats (CRISPR) is an adaptive immune system in eubacteria and archaebacteria that functions to counteract foreign nucleic acids, such as those of invading bacteriophages (Jinek *et al.* 2012). The CRISPR array encodes guide RNAs (gRNAs) that form complexes with Cas (CRISPR-associated) proteins. In a number of CRISPR/Cas systems, the Cas–gRNA complexes function as DNA endonuclease to cleave double-stranded DNA. The sequence of gRNA specifies the cleavage target. Accordingly, co-expression of a Cas protein and its cognate gRNA can introduce a DNA double-strand break (DSB) at a specific position in the genome. Cleaving the genome at a specific site is a critical process of genome editing. Because of the ease of specifying the target sequence, the CRISPR/Cas systems are being widely used for genome editing in a variety of organisms (Hsu *et al.* 2014).

A protospacer adjacent motif (PAM) is a short DNA sequence that flanks the target sequence defined by the gRNA and is required for the Cas-gRNA complex to recognize its target sequence (Sternberg *et al.* 2014). The nucleotide sequence of PAM is different from one CRISPR/Cas system to another. SpCas9 from *Streptococcus pyogenes* has a G-rich PAM (NGG) that follows the 3′ end of the target sequence (Jinek *et al.* 2012). AsCas12a from *Acidaminococcus* sp. has a T-rich PAM (TTTV) that precedes the 5′ end of the target sequence (Zetsche *et al.* 2015). SaCas9 from *Staphylococcus aureus* has a PAM with an intermediate GC content (NNGRRT) that flanks the 3′ end of the target sequence (Ran *et al.* 2015).

**Figure 1:**
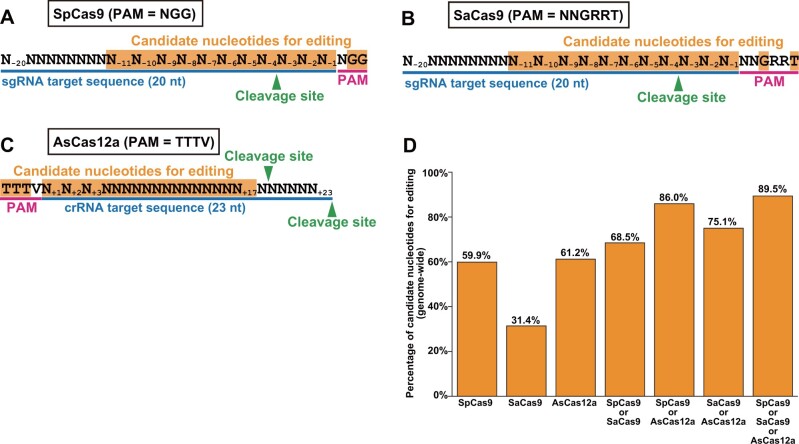
Estimation of editable nucleotides in the yeast genome. (A–C) Definition of candidate nucleotides for editing. PAM sequence for each CRISPR/Cas system is underlined with magenta. The target sequence in sgRNA/crRNA is underlined with blue. Candidate nucleotides for editing with each CRISPR/Cas system are highlighted in orange. Green arrowheads indicate cleavage sites. (D) Fraction of nucleotides in the reference genome editable with each and all possible combinations of the three CRISPR/Cas systems.

An absolute prerequisite for genome editing to insert a gene fragment, a single amino acid substitution, and a small indel to a specific site is a PAM in the vicinity of the target site. It, however, often happens that no appropriate PAM for a single CRISPR/Cas system is found in the region of interest. If multiple systems with different PAMs are available, it would theoretically become much easier to find a PAM and hence the target sequence to introduce a DNA DSB for gene fragment insertion, the introduction of single amino acid substitutions, and the introduction of small indels. Indeed, a simple calculation indicates the power of the combined use of SpCas9, SaCas9, and AsCas12a in genome editing of budding yeast *Saccharomyces cerevisiae* (see “Expansion of editable fraction of the yeast genome by combining three CRISPR/Cas systems” in the Results section and Figure 1).

These three CRISPR/Cas systems have already been implemented for the budding yeast (DiCarlo *et al.* 2013; Laughery *et al.* 2015; Generoso *et al.* 2016; Jessop-Fabre *et al.* 2016; Świat *et al.* 2017; Degreif *et al.* 2018; Verwaal *et al.* 2018). However, the vectors for these systems were independently developed in different laboratories. Consequently, the methods and design principles for genome-editing plasmid construction (*e.g.*, copy number, selection marker, promoter, cloning sites, and so on) are different from one to another. In practice, such differences often hamper seamless, stress-free use of the most suitable system to a given target site of interest. If all three systems can be used in a unified manner, then the genome-editing processes will be substantially accelerated.

Based on these theoretical and practical needs, we developed in this study a series of four backbone vectors under a unified design principle to seamlessly exploit SpCas9, SaCas9, and enAsCas12a in yeast genome editing. A single highly efficient method, Golden Gate Assembly, is applicable to construct genome-editing plasmids on these backbones. To facilitate the design of synthetic oligodeoxyribonucleotides (ODNs) required for the Golden Gate Assembly process, we developed a simple program that automatically calculates the ODN sequences corresponding to a given target sequence. We demonstrated that genome-editing plasmids thus constructed were efficient enough for routine use in gene knock-in at essential genes (median 52.1%), complete deletion of open reading frames (ORFs) (median 78.9%), and the introduction of single amino acid substitutions (median 79.2%).

## Materials and methods

### Yeast strains

Yeast strains used in this study are listed in Supplementary Table S1. All strains are derived from *S. cerevisiae* BY4741 or BY4742 (Brachmann *et al.* 1998). Standard culture media were used in this study (Guthrie and Fink 1991). Conventional gene deletion was performed using a PCR-based method (Longtine *et al.* 1998). Plasmids used for yeast strain construction are listed in Supplementary Table S2.

### Construction of backbone vectors for genome editing

ODNs used in this study are listed in Supplementary Table S3. All ODNs for plasmid construction were purchased from Sigma-Aldrich Japan (Tokyo, Japan) and Eurofins Genomics K. K. (Tokyo, Japan). The four backbone vectors for genome editing were constructed using the seamless cloning with HiFi DNA Assembly (E2621, New England Biolabs, Ipswich, MA, USA). Restriction enzymes used for plasmid construction were purchased from New England Biolabs. PCR fragments used for plasmid construction were amplified by Q5 DNA polymerase (M0491, New England Biolabs) according to manufacturer’s instruction. *Escherichia coli* competent cells NEB 5-alpha (C2987, New England Biolabs), NEB Stable (C3040, New England Biolabs), or Champion DH5α high (CC5202, SMOBIO Technology, Hsinchu City, Taiwan) were used for transformation to amplify and extract plasmids. Plasmids were extracted by FastGene Plasmid Mini Kit (FG-90502, Nippon Genetics, Tokyo, Japan). Plasmids used in this study are listed in Supplementary Table S2. The DNA sequence files of the backbone vectors for genome editing are available on our repository at GitHub (https://github.com/poccopen/Genome_editing_plasmid_for_budding_yeast).

### Selection of target sequences for genome editing

For the insertion of mNeonGreen-encoding sequence into the *CSE4* gene, we selected target sequences from the region encoding the unstructured N-terminal loop of Cse4 protein (Zhou *et al.* 2011; Yan *et al.* 2019). For the insertion of mScarlet-I-encoding sequence into the *CDC3* gene, we first performed secondary structure prediction by JPred4 (Drozdetskiy *et al.* 2015) of Cdc3 protein and then selected target sequences from the region encoding the N-terminal region with no predicted secondary structure.

For designing single guide RNAs (sgRNAs) for SpCas9 and SaCas9, CRISPRdirect (Naito *et al.* 2015) was used to select target sequences. For designing CRISPR RNAs (crRNAs) for enAsCas12a, CRISPOR (Concordet and Haeussler 2018) was used to select target sequences. Target sequences for genome editing used in this study are listed in Supplementary Table S4.

### Construction of genome-editing plasmids

All genome-editing plasmids were constructed using the seamless cloning with Golden Gate Assembly using NEB Golden Gate Assembly Kit (BsaI-HF v2) (E1601, New England Biolabs). The ODNs for Golden Gate Assembly were automatically designed with an in-house program.

### Yeast transformation for genome editing

Yeast transformation was carried out as described previously (Gietz and Woods 2002) with slight modifications. Yeast cells were cultured overnight in 2 mL of YPAD liquid medium (10 g/L Bacto Yeast Extract, #212750, Thermo Fisher Scientific, Waltham, MA, USA; 20 g/L Bacto Peptone, #211677, Thermo Fisher Scientific; 100 mg/L adenine sulfate, #01990-94, Nacalai tesque, Kyoto, Japan; and 20 g/L glucose, Nacalai tesque) at 25°C with shaking at 250 rpm. The 2-mL overnight culture was centrifuged and the supernatant was removed. The cell pellet was resuspended in 0.5 mL of 0.1 M lithium acetate solution (#127-01545, FUJIFILM Wako Chemicals, Osaka, Japan). The cell suspension was incubated at 30°C for 30 min. Fifty microliters of cell suspension were thoroughly mixed with 50 μL of 1 M lithium acetate, 50 μL of 1 M dithiothreitol (#14128-04, Nacalai tesque), 5 μL of Yeastmaker Carrier DNA (10 mg/mL, #630440, Takara Bio, Kusatsu, Japan), 1 μL of genome-editing plasmid (200–600 ng), 45 μL of PCR-generated donor fragment for gene fragment insertion (1–10 μg, typically 5 μg) or ORF deletion (2.5 μg), and 300 μL of polyethylene glycol 4000 (#11574-15, Nacalai tesque). PCR fragments were amplified by Q5 DNA polymerase (New England Biolabs) or KOD One (KMM-101, TOYOBO, Osaka, Japan) according to manufacturer’s instructions. The samples were incubated at 30°C for 45 min followed by a 15-min incubation at 42°C. After centrifugation and removal of supernatant, the cell pellets were resuspended with 50 μL of SC−Ura medium without carbon source (7.4 g/L Yeast nitrogen base without amino acids, #291940, Thermo Fisher Scientific; 855 mg/L CSM−Ura powder, DCS0161, FORMEDIUM, Hunstanton, UK; and 111 mg/L adenine sulfate, Nacalai tesque) and spread on a SCGal−Ura agar plate (20 g/L galactose, #075-00035, FUJIFILM Wako Chemicals; 6.7 g/L Yeast nitrogen base without amino acids, Thermo Fisher Scientific; 770 mg/L CSM−Ura powder, FORMEDIUM; 100 mg/L adenine sulfate, Nacalai tesque; and 20 g/L agar, #010-08725, FUJIFILM Wako Chemicals). The plates were incubated at 30°C for 4 days. The colonies were picked and streaked as patches on SCGal−Ura agar plates and then incubated at 30°C for 1–2 days, followed by colony PCR to check successful genome editing. Colony PCR was performed using Q5 DNA polymerase (New England Biolabs) or KOD One (TOYOBO) according to manufacturer’s instructions. The PCR-positive clones were cultured overnight in 2 mL of YPAD liquid medium. An aliquot (10 μL) of the overnight culture was spotted and streaked on a YPAD agar plate for single colony isolation (30°C for 2 days). Single colonies were picked and streaked on YPAD agar plate and SCDex−Ura agar plate (20 g/L glucose, #16806-25, Nacalai tesque; 6.7 g/L Yeast nitrogen base without amino acids, Thermo Fisher Scientific; 770 mg/L CSM−Ura powder, FORMEDIUM; 100 mg/L adenine sulfate, Nacalai tesque; and 20 g/L agar, #010-08725, FUJIFILM Wako Chemicals) to check the loss of the genome-editing plasmid. The Ura^−^ clones were re-examined by colony PCR to be successfully genome-edited. The colony PCR-positive Ura^−^ clones were used in the subsequent experiments.

### Plasmid extraction from yeast cells

Plasmids were extracted from yeast cells by Easy Yeast Plasmid Isolation Kit (#630467, Takara Bio) and transformed into *E. coli* competent cells (Champion DH5α high, SMOBIO Technology).

### Fluorescence microscopy and image processing

Image acquisitions of yeast cells were performed on a microscope (Ti-E, Nikon, Tokyo, Japan) with a 100× objective lens (CFI Apo TIRF 100XC Oil, MRD01991, Nikon), a sCMOS camera (ORCA-Fusion BT, C15440-20UP, Hamamatsu photonics, Hamamatsu, Japan), and a solid-state illumination light source (SOLA SE II, Lumencor, Beaverton, OR, USA). Image acquisition was controlled by NIS-Elements version 5.3 (Nikon). The binning mode of the camera was set at 2 × 2 (0.13 μm/pixel). Z-stacks were 13 × 0.3 μm. For imaging of Cse4-mNeonGreen, a filter set (LED-YFP-A, Semrock, Rochester, NY, USA) was used with excitation light power set at 20% and the exposure time set at 200 msec/frame. For imaging of Cdc3-mScarlet-I, a filter set (LED-TRITC-A, Semrock) was used with excitation light power set at 7% and exposure time set at 70 msec/frame. For DIC (differential interference contrast) image acquisition, the exposure time was set at 20 ms/frame. DIC images were captured only at the middle position of the Z-stacks.

Image processing and analysis were performed using Fiji (Schindelin *et al.* 2012). To generate 2-dimensional images of fluorescence channel from Z-stacks, background subtraction (sliding paraboloid radius set at 10 pixels with disabled smoothing) and maximum projection using 13 Z-slices were performed. Maximum projected fluorescence images and corresponding smoothed DIC images were superimposed. After global adjusting of brightness and contrast and cropping of the images, sequences of representative images were generated.

### Editable fraction of yeast genome with three CRISPR/Cas systems

*S. cerevisiae* reference genome sequence available at *Saccharomyces* genome database (SGD) (S288C strain, version R64-2-1, http://sgd-archive.yeastgenome.org/sequence/S288C_reference/genome_releases/S288C_reference_genome_R64-2-1_20150113.tgz) without mitochondrial genome and plasmid sequences were searched for PAMs (NGG for SpCas9, NNGRRT for SaCas9, and TTTV for AsCas12a). Both strands were included in the PAM search. After identifying the PAM sequence, nucleotides in a defined distance from the PAM were assigned as candidate nucleotides for editing. In genome editing, it is critical for a successfully edited target sequence in the genome not to be cleaved again by the Cas-gRNA complex bearing the gRNA corresponding to the original, unedited target sequence. We thus defined a nucleotide as a candidate for editing if its substitutions leading to mismatches with the gRNA can significantly reduce the efficiency of re-cleavage by the Cas-gRNA complex. For SpCas9 and SaCas9, the nucleotides at one to eleven nt away from the PAM and the nucleotides consisting the PAM were defined as the candidates based on previous reports (Anderson *et al.* 2015; Zheng *et al.* 2017; Tycko *et al.* 2018) (Figure 1, A and B). For AsCas12a, the nucleotides that are one to seventeen nt away from the PAM and the nucleotides consisting the PAM were defined as the candidates (Kim *et al.* 2016; Kleinstiver *et al.* 2016; Bin Moon *et al.* 2018) (Figure 1C). Degenerate nucleotides in the PAMs (*i.e.*, N, R, and V) were excluded from the calculation (Figure 1, A–C). The total number of candidate nucleotides for editing is summarized in Supplementary Table S5 and Figure 1D.

## Results

### Expansion of editable fraction of the yeast genome by combining three CRISPR/Cas systems

We evaluated the potentials of three well-established CRISPR/Cas systems with distinct PAMs, namely SpCas9 from *S. pyogenes* (Jinek *et al.* 2012), SaCas9 from *S. aureus* (Ran *et al.* 2015), and enhanced AsCas12a (enAsCas12a) derived from *Acidaminococcus* sp. (Kleinstiver *et al.* 2019), in editing the budding yeast genome. For each system, we calculated the number of “editable” nucleotides in the reference genome sequence of the budding yeast strain, S288C (see “Editable fraction of yeast genome with three CRISPR/Cas systems” in the *Materials and methods* section). This simple simulation indicated that while SpCas9, SaCas9, and AsCas12a can target 59.9%, 31.4%, and 61.2% of the genome, respectively, their combined use can cover as much as 89.5% (Figure 1D, Supplementary Table S5). Based on this simulation, we decided to develop a backbone vector series sharing a single design principle to enable the seamless use of the three CRISPR/Cas systems to expand the repertoire of editable genes.

### Design of backbone vectors for yeast genome editing

**Figure 2:**
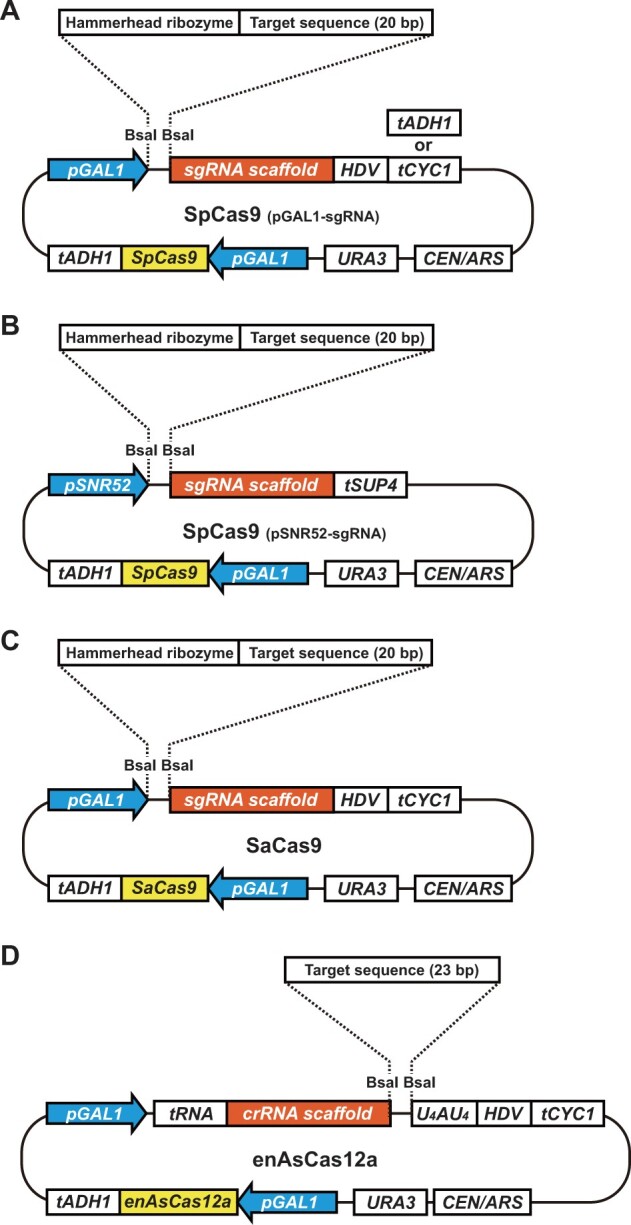
Schematic representation of backbone vectors for genome editing. (A) SpCas9 + pGAL1-sgRNA system. (B) SpCas9 + pSNR52-sgRNA system. (C) SaCas9 system. (D) enAsCas12a system. *pGAL1*, *GAL1* promoter; *pSNR52*, *SNR52* promoter; *tADH1*, *ADH1* terminator; *tCYC1*, *CYC1* terminator; *tSUP4*, *SUP4* terminator; HDV, HDV ribozyme; *U_4_AU_4_*, 9-mer encoding “UUUUAUUUU” for improvement of genome-editing efficiency; *URA3*, *URA3* marker cassette; *CEN/ARS*, centromere and autonomously replicating sequence

In the design of the vector series, we defined the following three requirements: (1) both Cas protein and sgRNA/crRNA are encoded on a single plasmid, (2) expression of Cas protein and/or sgRNA/crRNA can be artificially induced, and (3) target sequence of sgRNA/crRNA can be incorporated using the Golden Gate Assembly (Engler *et al.* 2008). Fulfilling these three requirements, we developed four backbone centromeric plasmid vectors marked with *URA3* for the three CRISPR/Cas systems (Figure 2). Note that enAsCas12a was used instead of AsCas12a because of its improved activity at lower temperatures suitable to grow budding yeast cells (Kleinstiver *et al.* 2019).

As an inducible promoter, we use the well-characterized *GAL1* promoter because it is actively repressed by glucose and strongly activated by galactose in the absence of glucose. Cas-encoding genes on the four vectors are placed under the control of the *GAL1* promoter. Similarly, sgRNA/crRNA precursors on three vectors are controlled by the *GAL1* promoter.

The Golden Gate Assembly uses type IIS restriction enzymes such as BsaI and BbsI (Engler *et al.* 2008). Our vector series harbors two BsaI recognition sites for Golden Gate Assembly. Because the target sequence lies at the 5′ terminal side of the sgRNA scaffold for SpCas9 and SaCas9, one BsaI site is placed just downstream of the *GAL1* or *SNR52* promoter, and the other site is placed just upstream of the sgRNA scaffold sequence (Figure 2, A–C). In the case of enAsCas12a, the target sequence is located at the 3′ terminal side of the crRNA scaffold. Accordingly, one BsaI site is placed just downstream of the crRNA scaffold, and the other site is placed further downstream (Figure 2D).

Extra sequences at the 5′ and 3′ ends of sgRNA often compromise the efficiency of genome editing and hence should be adequately trimmed. To remove the 5′ extra sequence, a hammerhead ribozyme is inserted at the beginning of the sgRNA-containing transcript from the three Cas9 vectors (Figure 2, A–C). To remove the 3′ extra sequence in the transcripts driven by *GAL1* promoter, the hepatitis delta virus (HDV) ribozyme is inserted to the 3′ side of the sgRNA scaffold (Figure 2, A and C). For the other vector using *SNR52* promoter driven by RNA polymerase III, *SUP4* terminator is used to define the 3′ end of transcript (Figure 2B). In the enAsCas12a vector, the crRNA is preceded and followed by tRNA(Gly) (Zhang *et al.* 2019) and the HDV ribozyme, respectively, for the removal of extra sequences (Figure 2D). Furthermore, a sequence encoding “UUUUAUUUU” is inserted between the second BsaI site (*i.e.*, 3′ end of the crRNA) and the HDV ribozyme, because this 9-mer sequence was demonstrated to increase the efficiency of genome editing (Bin Moon *et al.* 2018).

### A program to design ODNs for Golden Gate Assembly

To construct a genome-editing plasmid, a pair of ODNs corresponding to its target sequence must be synthesized so that they include 4-nt sequences compatible with the backbone vectors. Furthermore, a hammerhead ribozyme compatible with each target sequence must be designed and included in the ODNs (Supplementary Figure S1A). To facilitate this complicated process without the risk of human errors, we created a simple program that automatically calculates the ODNs for a given target sequence (Supplementary Figure S1B). Upon entering a target sequence with its name followed by the selection of a backbone vector, the program readily provides ODN sequences to be synthesized for the Golden Gate Assembly of a genome-editing plasmid on the selected backbone vector. The program is available from our repository on GitHub (https://github.com/poccopen/Genome_editing_plasmid_for_budding_yeast).

### Application example 1: gene insertion by SpCas9 and enAsCas12a systems

In budding yeast, while the C-terminal tagging of an essential gene can be performed easily in a single-step procedure (Longtine *et al.* 1998), the insertion of a DNA fragment to an essential gene at a location other than its C-terminus requires multiple steps and more extended time. Genome-editing using CRISPR/Cas systems can simplify the gene fragment insertion processes. As an application example of the CRISPR/Cas systems, we attempted to insert a fluorescent protein gene into an internal portion of essential genes. We chose the *CSE4* gene and the *CDC3* gene as our targets of fluorescent protein gene insertion. The *CSE4* gene encodes a centromere-specific histone H3 variant, Cse4 (Stoler *et al.* 1995). It was reported that when a fluorescent protein is fused at the C-terminus of Cse4, the cells show temperature sensitivity (Wisniewski *et al.* 2014). In contrast, when the fluorescent protein is inserted into the unstructured N-terminal loop of Cse4 (Zhou *et al.* 2011; Yan *et al.* 2019), the cells grow normally at a higher temperature (Wisniewski *et al.* 2014). The *CDC3* gene encodes one of the septin proteins, which form a ring structure along the bud neck (Caviston *et al.* 2003). It was shown that when a fluorescent protein is fused to the C-terminus of Cdc3, the localization of Cdc3 protein becomes abnormal, leading to a morphological defect (Huh *et al.* 2003; Dubreuil *et al.* 2019). In contrast, when a fluorescent protein is inserted into an N-terminal loop of Cdc3, the tagged Cdc3 protein correctly localizes at the bud neck, and the cells grow normally without showing any morphological defect (Caviston *et al.* 2003).

**Figure 3:**
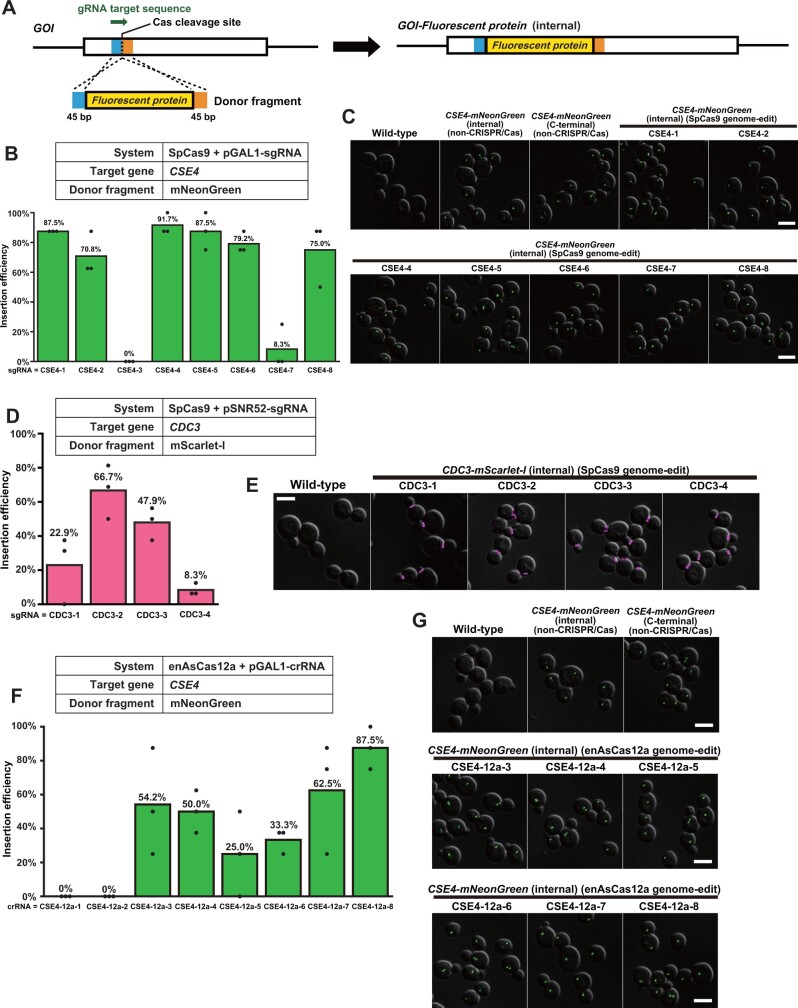
Gene fragment insertion into essential genes using the SpCas9 and enAsCas12a systems. (A) Gene fragment insertion process by genome editing using CRISPR/Cas systems at a target sequence colored green. *GOI*, gene of interest. A fluorescent protein gene to be inserted is colored yellow. The 45-bp regions used as the 5′- and 3′-homology arms are colored blue and orange, respectively. (B) The efficiency of *mNeonGreen* gene fragment insertion into the essential gene *CSE4* using the SpCas9 + pGAL1-sgRNA system at 8 target sequences. Green bars indicate the average insertion efficiency over three experiments (n = 24 in total). Black dots show the insertion efficiency of each experiment (n = 8 for each). (C) Representative images of the wild-type cells and the *CSE4-mNeonGreen* cells. Images are composed by the superimposition of DIC images (grayscale) and mNeonGreen fluorescent images (green). The target sequence names are shown above the images. Scale bar, 5 μm. (D) The efficiency of *mScarlet-I* gene fragment insertion into the essential gene *CDC3* using the SpCas9 + pSNR52-sgRNA system at 4 target sequences. Magenta bars indicate the average insertion efficiency over three experiments (n = 48 in total). Black dots show the insertion efficiency of each experiment (n = 16 for each). (E) Representative images of the wild-type cells and the genome-edited *CDC3-mScarlet-I* cells. Images are composed by the superimposition of DIC images (gray scale) and mScarlet-I fluorescent images (magenta). The target sequence names are shown above the images. Scale bar, 5 μm. (F) The efficiency of *mNeonGreen* gene fragment insertion into the essential gene *CSE4* using the enAsCas12a + pGAL1-crRNA system at 8 target sequences. Green bars indicate the average insertion efficiency over three experiments (n = 24 in total). Black dots show the insertion efficiency of each experiment (n = 8 for each). (G) Representative images of the wild-type cells and the *CSE4-mNeonGreen* cells. Images are composed by the superimposition of DIC images (gray scale) and mNeonGreen fluorescent images (green). The target sequence names are shown above the images. Scale bar, 5 μm.

As an application example of the SpCas9 + pGAL1-sgRNA system, we attempted to insert a gene fragment encoding mNeonGreen, a bright yellow–green fluorescent protein (Shaner *et al.* 2013), into the N-terminal loop of Cse4. We designed 8 target sites in the genic region encoding the N-terminal loop of Cse4. To insert the *mNeonGreen* gene fragment into these sites, we prepared donor PCR fragments harboring 45-bp homology arms at both termini (Figure 3A). For each of the 8 target sites, the yeast cells were co-transformed with the corresponding genome-editing plasmid and the donor PCR fragment. The transformants formed a mixture of large and small colonies (Supplementary Figure S2A). The gene fragment insertion efficiency of the small and large colonies was evaluated by a PCR assay using primer sets flanking the insertion position. For the target sequence CSE4-1, small colonies showed a significantly higher insertion efficiency (87.5%, *n* = 24, or 8 colonies in each of 3 biological replicates) than large colonies (4.2%, *n* = 24, or 8 colonies in each of 3 biological replicates) (Supplementary Figure S2B).

Observing the heterogeneity in colony size, we hypothesized that loss of genome-editing function results in loss of cell cycle arrest induced by DNA DSB and its repair and leads to the formation of large colonies. It was likely that intramolecular recombination between the two *GAL1* promoters led to the loss of SpCas9 expression cassette (Supplementary Figure S2C). To test this hypothesis, we analyzed the structure of the genome-editing plasmids in the cells forming large colonies by restriction enzyme digestion and PCR. All the 24 plasmids derived from large colonies showed the structural change consistent with the predicted deletion caused by recombination between the two *GAL1* promoters (Supplementary Figure S2, D and E). Based on these results, we picked only small colonies in the subsequent genome-editing experiments with plasmids harboring two *GAL1* promoters.

Colony size and the number of colonies were analyzed for the plates shown in Supplementary Figure S2 using image analysis (Supplementary Figure S3). While the number of small colonies is highly variable depending on the target sequence, the frequency of appearance of large colonies is almost constant among the eight plates (Supplementary Figure S3, B and C). This result shows that it is likely that the frequency of intramolecular recombination through two *GAL1* promoter sequences is stable. There is a strong negative correlation between the number of small colonies and the insertion efficiency (Supplementary Figure S3F). This result suggests that the gRNA efficacy, which is reflected in the viability of the cells, is a major determinant of the success rate of the genome-editing. In contrast, a significant correlation was not observed between the number of large colonies and the insertion efficiency (Supplementary Figure S3E). This result suggests that the intramolecular recombination takes place independently of the genome-editing processes. Based on the colony numbers of the cells (CSE4-3, showing the highest viability among the eight conditions), the frequency of intramolecular recombination is estimated to be no more than 4.6% (65/1407) (Supplementary Figure S3, A and B).

Using CSE4-1 as a model target sequence, we also investigated the relationship between the length of homology arms and the insertion efficiency. We used PCR fragments harboring 4 different homology arm lengths (15-, 25-, 35-, and 45-bp) for genome editing. There was a positive correlation between homology arm length and insertion efficiency (Supplementary Figure S4). A similar positive correlation has been reported between homology arm length and genome-editing efficiency in fission yeast (Hayashi and Tanaka 2019). When a donor PCR fragment harboring 15-bp homology arms was used, no gene insertion was observed (Supplementary Figure S4). Based on these results, PCR fragments harboring 45-bp homology arms were used in the subsequent genome-editing experiments for gene insertion.

Among the 8 target sequences (Supplementary Table S4), the gene insertion efficiency varied from 0% to 91.7% (*n* = 24 for each target sequence) (Figure 3B, Supplementary Table S4). Clones with successful gene insertions were obtained for 7 out of the 8 target sequences.

We investigated the phenotype of the successfully genome-edited cells. In all the genome-edited clones tested, the mNeonGreen fluorescence signal was localized as a single spot or a pair of spots in each cell (Figure 3C and Supplementary Figure S5A). The localization pattern of the mNeonGreen signal was indistinguishable between the genome-edited cells and the cells generated using the conventional method to harbor mNeonGreen at the Cse4 N-terminal loop (Supplementary Figure S5A). These two genome-modified cells showed comparable growth at 37°C with the wild-type cells, whereas those harboring mNeonGreen at the C-terminus of Cse4 did not (Supplementary Figure S5B).

As an example of the use of SpCas9 + pSNR52-sgRNA system, we attempted to insert a gene fragment encoding a bright red fluorescent protein, mScarlet-I (Bindels *et al.* 2017), into an N-terminal region of Cdc3 predicted to lack any secondary structure. We designed 4 target sequences in the genic region corresponding to the N terminal region. To insert the mScarlet-I gene fragment, we prepared donor PCR fragments harboring 45-bp homology arms at both termini (Figure 3A). The transformation of yeast cells with a genome-editing plasmid and a corresponding donor PCR fragment resulted in a mixture of large and small colonies (Supplementary Figure S6A). However, for all the 4 target sequences, there was no statistically significant difference in insertion efficiency between the small and large colonies (Supplementary Figure S6B).

The gene insertion efficiency varied from 8.3% to 66.7% among the 4 target sequences (*n* = 48 for each target sequence) (Figure 3D). In all the genome-edited clones examined, mScarlet-I fluorescent signal was localized as a ring structure at the bud neck (Figure 3E and Supplementary Figure S6C). None of the 12 genome-edited clones (3 clones for each target sequence) exhibited morphological defect (Supplementary Figure S6C). All the genome-edited clones (4 clones for each target sequence) showed comparable growth at 37°C with the wild-type cells (Supplementary Figure S6D).

As an application example of enAsCas12a + pGAL1-crRNA system, we attempted to insert a gene fragment encoding mNeonGreen to the genic regions encoding the N-terminal loop of Cse4, as we did above (Figure 3, B and C). We designed 8 target sequences and prepared donor PCR fragments harboring 45-bp homology arms at both termini (Figure 3A). The gene insertion efficiency varied from 0% to 87.5% among the 8 target sequences (*n* = 24 for each target sequence) (Figure 3F). Clones with successful gene insertions were obtained for 6 out of the 8 target sequences (Figure 3F). In all genome-edited clones, the mNeonGreen fluorescence signal was localized as a single spot or a pair of spots in each cell (Figure 3G and Supplementary Figure S7A), and the growth at 37°C was comparable to the wild-type cells (Supplementary Figure S7B). These results were consistent with those obtained for the cells generated using the conventional approach and the SpCas9 + pGAL1-sgRNA system.

### Application example 2: complete ORF deletion by SaCas9 system

**Figure 4:**
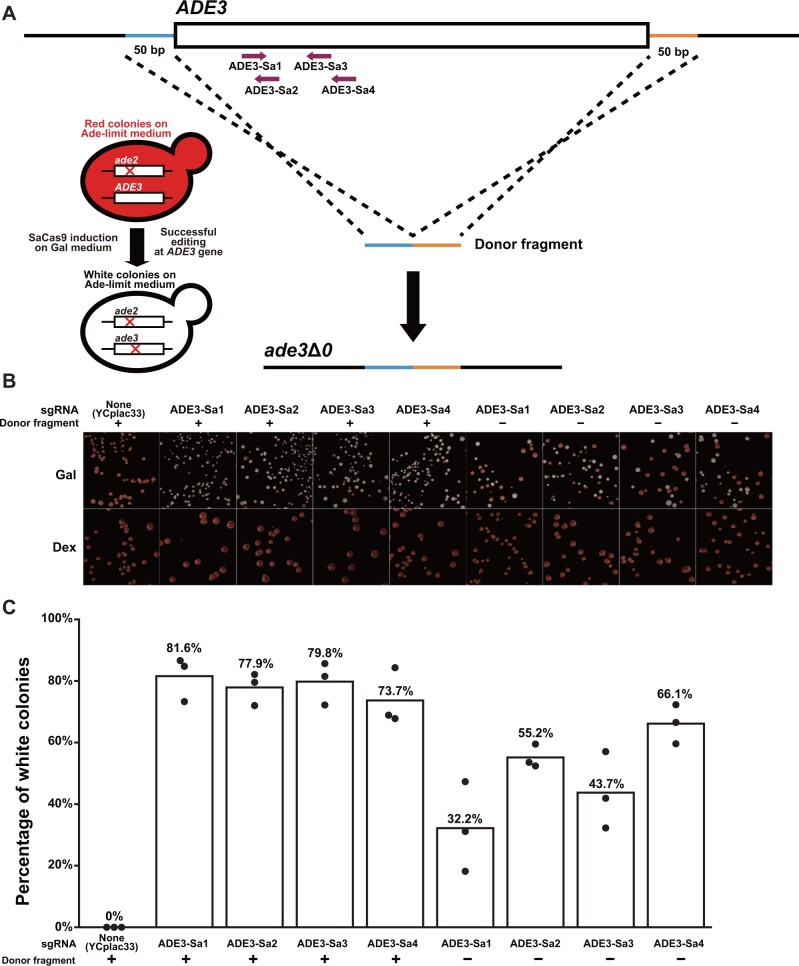
Complete deletion of *ADE3* ORF using the SaCas9 system. (A) Process for deleting the entire *ADE3* ORF and converting colony color. The dark magenta arrows represent the positions of the target sequences for SaCas9. The 50-bp regions used as the 5′- and 3′-homology arms of the 100-bp donor PCR fragment are colored blue and orange, respectively (schematic not proportional to actual size). While *ade2*Δ cells accumulate red pigments on adenine-limited medium, *ade2*Δ*ade3*Δ cells fail to do so and form white colonies. (B) Representative images of colonies after transformation of the genome-editing plasmids with or without the donor PCR products on adenine-limited galactose-containing medium (top, Gal) or adenine-limited glucose-containing medium (bottom, Dex). The target sequences used for genome editing are shown above the panels. (C) The efficiency of colony color conversion. Each white bar indicates the percentage of white colonies in each experimental condition shown at the bottom. The value is the average of 3 biological replicates indicated by black dots.

As an example of the use of SaCas9 + pGAL1-sgRNA system, we attempted to delete an entire ORF. We chose the *ADE3* gene as a target of complete ORF deletion. The *ADE3* gene encodes C1-tetrahydrofolate synthase, an enzyme required for adenine biosynthesis (McKenzie and Jones 1977). The cells lacking the *ADE2* gene encoding phosphoribosylaminoimidazole carboxylase, another enzyme in the adenine biosynthesis pathway, form red colonies by accumulating intermediate metabolites of red color (Hieter *et al.* 1985). When the *ADE3* gene is deleted in the cells lacking *ADE2*, colony color returns to white because of the loss of accumulation of the red metabolites (Koshland *et al.* 1985). We attempted to delete the *ADE3* ORF in an *ade2*Δ strain and convert colony color from red to white (Figure 4A). We selected 4 target sequences in the ORF, constructed the corresponding genome-editing plasmids, and used them to transform the *ade2*Δ cells with or without a 100-bp donor PCR fragment composed of the 5′- and 3′-flanking sequences of the ORF (Figure 4A). The transformation with the genome-editing plasmids resulted in the formation of white colonies on galactose-containing plates (Figure 4B, top row). Transformation with a control plasmid, YCplac33 vector with no expression of Cas protein and sgRNA, failed to form white colonies (Figure 4B). Even when the genome-editing plasmids were used, white colonies did not appear among the transformants on glucose-containing plates (Figure 4B, bottom row). These results indicated that the galactose-inducible SaCas9 system worked as we expected.

**Figure 5:**
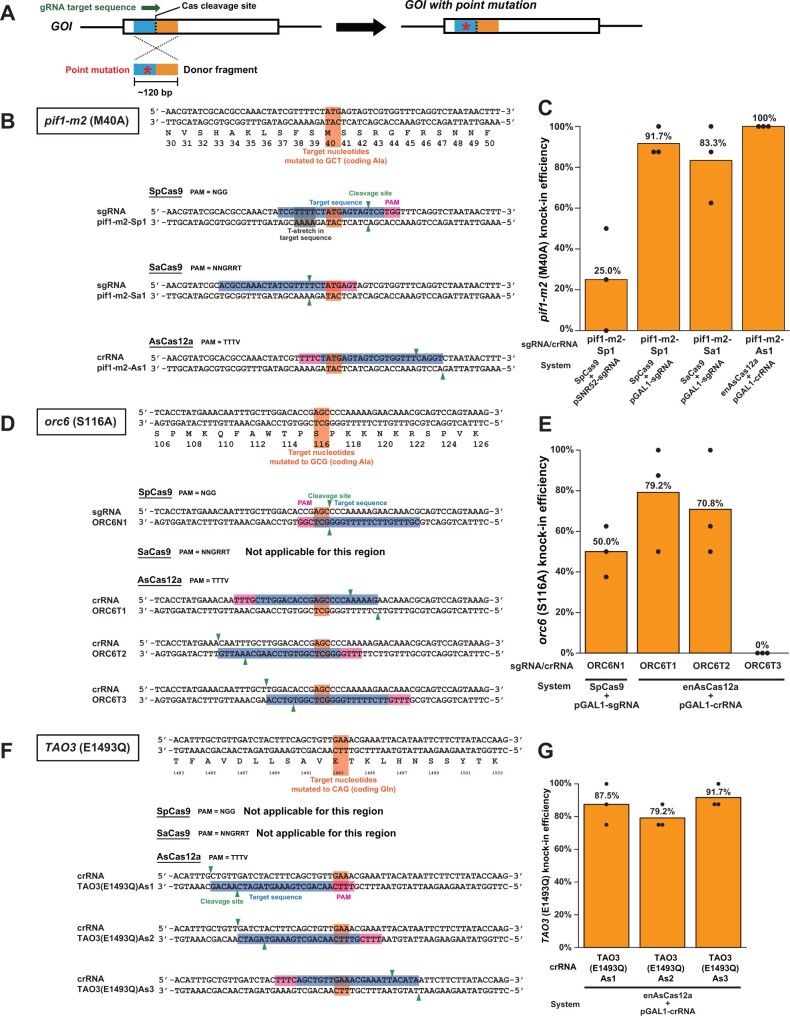
Introduction of single amino acid substitutions using the SpCas9, SaCas9, and enAsCas12a systems. (A) Process to introduce a single amino acid substitution in the product of a gene of interest (*GOI*) using CRISPR/Cas systems. The target sequence is colored green. The 5′- and 3′-homology arms are colored blue and orange, respectively. A red asterisk indicates the point mutation in the donor fragment. (B) Design of sgRNA/crRNAs to generate *pif1-m2* (M40A) allele. The M40 codon is colored orange. PAM motifs are colored pink. Target sequences for sgRNA/crRNAs are colored blue. Green triangles indicate Cas cleavage sites. A T_4_ stretch in a target sequence (pif1-m2-Sp1) is colored gray. (C) The knock-in efficiency of the M40A substitutions into the *PIF1* gene using CRISPR/Cas systems. Orange bars indicate the average insertion efficiency over three experiments (*n* = 24 in total). Black dots show the insertion efficiency of each experiment (*n* = 8 for each). (D) Design of sgRNA/crRNAs to generate *orc6* (S116A) allele. The S116 codon is colored orange. PAM motifs are colored pink. Target sequences for sgRNA/crRNAs are colored blue. Green triangles indicate Cas cleavage sites. (E) The knock-in efficiency of the S116A substitutions into the *ORC6* gene using CRISPR/Cas systems. Orange bars indicate the average insertion efficiency over three experiments (*n* = 24 in total). Black dots show the insertion efficiency of each experiment (*n* = 8 for each). (F) Design of crRNAs to generate *TAO3* (E1493Q) allele. The E1493 codon are colored orange. PAM motifs are colored pink. Target sequences for crRNAs are colored blue. Green triangles indicate enAsCas12a cleavage sites. (G) The knock-in efficiency of the E1493Q substitutions into the *TAO3* gene using the enAsCas12a-based system. Orange bars indicate the average insertion efficiency over three experiments (*n* = 24 in total). Black dots show the insertion efficiency of each experiment (*n* = 8 for each).

When the cells were transformed with a genome-editing plasmid and the donor PCR fragment for the ORF deletion, the proportion of white colonies on galactose-containing plates was in the range of 73.7–81.6% (Figure 4C). The formation of white colonies does not necessarily indicate complete deletion of *ADE3* ORF, as small insertion or deletion (indel) could also result in the loss of Ade3 function. We performed a PCR assay to distinguish the complete deletion of *ADE3* ORF from small indels (Supplementary Figure S8A). PCR products consistent with complete deletion of *ADE3* ORF were obtained in all the 32 white colonies examined (8 colonies for each target sequence) (Supplementary Figure S8B, top). We then checked the sequences of these PCR products and confirmed the complete loss of *ADE3* ORF (Supplementary Figure S9).

We also attempted to knock out the *ADE3* gene through nonhomologous end joining. For this purpose, we transformed the yeast cells solely with the genome-editing plasmids. In this case, loss of the *ADE3* gene function should be attributable to frameshift mutations caused by indels in the vicinity of SaCas9 cleavage site. The proportion of white colonies on galactose-containing plates ranged from 32.2% to 66.1%, which is lower than that of the cells co-transformed with the genome-editing plasmids and the donor PCR fragment (73.7%–81.6%) (Figure 4C). We performed a PCR assay to exclude the possibility of large deletions (Supplementary Figure S8, A and B, bottom). Sequencing of these PCR products confirmed the presence of small indels (1–2 bp) in the vicinity of the expected SaCas9 cleavage sites (Supplementary Figure S10).

### Application example 3: introduction of single amino acid substitutions by SpCas9, SaCas9, and enAsCas12a systems

As another example of the use of the CRISPR/Cas systems, we attempted to introduce single amino acid substitutions and chose three target genes, *PIF1*, *ORC6*, and *TAO3*.

*PIF1* encodes a DNA helicase which facilitates DNA synthesis during break-induced replication (Chung *et al.* 2010). Pif1 functions in the nucleus and mitochondria (Schulz and Zakian 1994). The nuclear and mitochondrial functions of *PIF1* can be separated by mutations (Schulz and Zakian 1994). *PIF1* has two initiation codons which determine localization of the Pif1 protein: translation starting from the first initiation codon (M1) and the second one (M40) leads to the production of Pif1 protein bearing and lacking the N-terminal mitochondrial targeting signal, respectively. Accordingly, *pif1-m1* (M1A) allele encodes only the protein isoform that starts from M40 to lack the targeting signal and is hence incapable of mitochondrial localization. In contrast, *pif1-m2* (M40A) allele encodes only the isoform that starts from M1 to bear the targeting signal and is hence imported to mitochondria. In this study, we attempted to generate the *pif1-m2* (M40A) allele using the CRISPR/Cas systems. For the introduction of the M40A substitution, only a single sgRNA/crRNA can be designed for each of the CRISPR/Cas (Figure 5B). We thus constructed a total of four genome-editing plasmids using the four backbone vectors (Figure 2). We prepared a donor PCR fragment containing mutations relevant to M40A substitution (Figure 5A). For each of the target sites, the yeast cells were co-transformed with the corresponding genome-editing plasmid and the donor PCR fragment. The knock-in efficiency was evaluated by a pair of PCR assays using primer sets specific for the M40A mutant allele and the wild-type M40M allele (Supplementary Figure S11). We also checked the sequence of the region and confirmed the introduction of the M40A substitution (Supplementary Figure S12). Among the four systems, the knock-in efficiency varied from 25.0% to 100% (*n* = 24 for each target sequence) (Figure 5C). Compared to the systems using the pGAL1-sgRNA/crRNA systems (83.3%–100%), the SpCas9 + pSNR52-sgRNA system showed a lower efficiency (25.0%). It is possible that the T_4_ stretch in the target sequence (Figure 5B) can lead to a pause or premature termination of transcription of the sgRNA by RNA polymerase III (Arimbasseri and Maraia 2015).

*ORC6* encodes a subunit of the origin recognition complex required to start DNA replication (Li and Herskowitz 1993). It has been reported that mutations causing substitution of Ala for Ser-116 of Orc6, a target site of cyclin-dependent kinases, increases the frequency of re-replication under a specific genetic background (Richardson and Li 2014). We attempted to generate *orc6* (S116A) allele using CRISPR/Cas systems. For the introduction of the S116A substitution, one sgRNA, no sgRNA, and three crRNA can be designed for SpCas9, SaCas9, and AsCas12a, respectively (Figure 5D). We obtained the co-transformants of the four pairs of the genome-editing plasmid and the donor PCR fragment and analyzed them with PCR and sequencing (Supplementary Figures S13 and S14). The knock-in efficiency varied from 0% to 79.2% (*n* = 24 for each target sequence) (Figure 5E). Clones with successful gene insertions were obtained for 3 out of the 4 target sequences.

*TAO3* encodes a protein involved in apical bud growth and cell morphogenesis. Intriguingly, *TAO3* (E1493Q) allele was identified as one of the quantitative trait loci affecting the sporulation efficiency (Deutschbauer and Davis 2005). While the S288C strain and its derivative BY4741 strain have the *TAO3* (E1493E) allele and show a very low sporulation efficiency, the SK1 strain has the *TAO3* (E1493Q) allele and shows a very high sporulation efficiency. The introduction of the *TAO3* (E1493Q) allele in the S288C cells increased the sporulation efficiency. In this study, we attempted to generate the *TAO3* (E1493Q) allele using the CRISPR/Cas systems. Notably, the E1493Q substitution was found to be achievable only with AsCas12a (Figure 5F). For each of the three target sites, the yeast cells were co-transformed with the corresponding genome-editing plasmid and the donor PCR fragment containing the relevant mutations, and the co-transformants were examined with PCR and sequencing (Supplementary Figures S15 and S16). Among the three crRNAs, the knock-in efficiency varied from 79.2% to 91.7% (*n* = 24 for each target sequence) (Figure 5G). Clones with successful gene insertions were obtained for 3 out of the 3 target sequences.

## Discussion

Here, we reported a series of vectors for yeast genome editing using three different CRISPR/Cas systems, namely SpCas9, SaCas9, and enAsCas12a (Figure 2). Because the three systems have distinct PAMs, their combined use expands the editable fraction of the yeast genome, as indicated by our simulation (Figure 1). To facilitate the seamless use of these systems, we constructed a vector series under a unified design principle. First, all the vectors harbor *URA3* marker and *GAL1* promoter, thus sharing the media required for their use. Accordingly, if a single certain system fails to edit a region of interest, one can readily switch to another system without preparing any additional medium. Second, all the vectors are compatible with the highly efficient Golden Gate Assembly, thus making the construction step virtually free from failure. Furthermore, a dedicated program is developed to design ODNs for Golden Gate Assembly of individual genome-editing plasmids on these backbone vectors. Target search with CRISPRdirect (Naito *et al.* 2015) and CRISPOR (Concordet and Haeussler 2018) followed by ODN design with this program would thus streamline the entire process to design genome-editing plasmids.

The realistic schedule of genome editing described in this study is summarized in Table 1. The entire process from designing a genome-editing plasmid to obtaining genome-edited strains can be completed within 2 weeks. This period is substantially shorter than the one required for the traditional yeast genetics approach, especially, in the case of inserting a gene fragment into an essential gene. For instance, when using the own promoter of an essential gene, the traditional approach includes the construction of a cover plasmid carrying the wild-type allele of the essential gene, transformation of the cover plasmid, disruption of the genomic copy of the essential gene, introduction of an adequately modified allele, and the curing of the cover plasmid, thus taking at least 17 days or, more realistically, >20 days.

**Table 1:** Schedule of genome editing

Day	Procedures
1	Select target sequences; design and order ODNs
2	
3	Receive ODNs; perform Golden Gate Assembly; transform *E. coli* cells
4	Inoculate *E. coli* clones for plasmid extraction; inoculate yeast cells for genome-editing transformation
5	Extract plasmid from *E. coli* clones; prepare donor fragments with PCR; transform yeast cells
6	
7	
8	
9	Pick up yeast colonies
10	Perform yeast colony PCR; inoculate yeast cells in YPAD
11	Spread yeast cells for single colony isolation
12	
13	Pick up single yeast colonies
14	Perform yeast colony PCR

It is intriguing to note that the period for genome editing can be further shortened with these plasmids. In this study, we used galactose to strongly activate *GAL1* promoter at the expense of substantially compromised growth compared to that in the presence of glucose, the ideal carbon source for *S. cerevisiae*. Notably, the artificial transcription factor GEV (*i.e.*, a fusion protein composed of Gal4 DNA-binding domain, estrogen receptor, and VP16) can activate the *GAL1* promoter upon estradiol addition in glucose media (Hickman *et al.* 2011). We thus expect that a GEV-bearing strain reconciles efficient induction and rapid growth, thereby further shortening the period required for genome editing using these vectors.

We examined the performance of genome-editing plasmids using these backbone vectors in our attempts for the insertion of gene fragments to essential genes (Figure 3), the complete deletion of an ORF (Figure 4), and the introduction of single amino acid substitutions (Figure 5). In the case of gene fragment insertion, successfully genome-edited cells were obtained for 17 out of 20 target sequences examined (Figure 3, B, D, and F), and the insertion efficiency exceeded 50% for 11 target sequences. In the case of complete ORF deletion, the efficiency was larger than 70% for all the 4 target sequences tested (Figure 4C). We also examined the growth of genome-edited clones at 30°C and 37°C (Supplementary Figures S5B, S6D, and S7B). None of the 55 clones showed temperature-sensitive growth. In the case of the introduction of single amino acid substitutions, successfully genome-edited cells were obtained for 10 out of 11 sgRNAs/crRNAs examined (Figure 5, C, E, and G), and the knock-in efficiency exceeded 50% for 9 target sequences. These results proved the practical utility of the vector series developed in this study.

For one of the target genes for the introduction of single amino acid substitutions [*TAO3* (E1493Q)], it is impossible to design sgRNAs for SpCas9 and SaCas9 due to the lack of the cognate PAMs in the vicinity of the target site (Figure 5F). Accordingly, the genome-editing was only possible and successfully attained with the enAsCas12a system (Figure 5G). This result demonstrates the value of having all three systems available as a vector series constructed in a unified design principle.

We should refer to a practical rule of thumb for successful genome editing using the three backbone vectors bearing two *GAL1* promoters. When using these vectors, intramolecular recombination between the two promoters tends to lead to the formation of large colonies with low efficiency of genome editing (Supplementary Figure S2). We thus recommend the users of these vectors to simply discard large colonies and select small ones for further analyses because the latter showed significantly higher genome-editing efficiency than the former (Supplementary Figure S2). While the single *GAL1* promoter plasmid also led to heterogeneity in colony size, no difference in genome-editing efficiency was observed between large and small colonies (Supplementary Figure S6).

Our application examples included the insertion of fluorescent proteins into positions that are neither N- nor C-end of the essential proteins Cse4 and Cdc3 (Figure 3). Tagging at inappropriate sites of these proteins was reported to induce temperature-sensitive growth and/or morphological defects. To avoid the adverse effects of inserting a fluorescent protein on the recipient protein folding, we took a strategy to select an insertion site from regions demonstrated or predicted to have no secondary structure. All the proteins thus fluorescently tagged, including those using previously unvalidated sites, showed physiological localization, and the cells thus modified exhibited neither temperature-sensitive growth nor abnormal morphology. These results suggest the general utility of our strategy.

Taken together, the backbone vectors and the program developed in this study would provide a versatile toolbox to facilitate various types of genome manipulation in *S. cerevisiae*, including those challenging to perform with conventional techniques in yeast genetics.

## Data availability

The four backbone vectors are available from NBRP Yeast Resource Center (https://yeast.nig.ac.jp/yeast/). The source codes of programs for ODN design and PAM search are available from our repository at GitHub (https://github.com/poccopen/Genome_editing_plasmid_for_budding_yeast). Other strains and plasmids are available upon request. The authors state that all data necessary for confirming the conclusions presented here are represented fully within the article.

Supplementary material is available at *G3* online.

## Supplementary Material

jkab304_Supplementary_Figures

jkab304_Supplementary_Tables

## References

[jkab304-B1] Anderson EM , Haupt A, Schiel JA, Chou E, Machado HB, et al 2015. Systematic analysis of CRISPR-Cas9 mismatch tolerance reveals low levels of off-target activity. J Biotechnol. 211:56–65. doi:10.1016/j.jbiotec.2015.06.427. 26189696 10.1016/j.jbiotec.2015.06.427

[jkab304-B2] Arimbasseri AG , Maraia RJ. 2015. Mechanism of transcription termination by RNA polymerase III utilizes a non-template strand sequence-specific signal element. Mol Cell. 58:1124–1132. doi:10.1016/j.molcel.2015.04.002. 25959395 10.1016/j.molcel.2015.04.002 PMC4475470

[jkab304-B3] Bin Moon S , Lee JM, Kang JG, Lee N-E, Ha D-I, et al 2018. Highly efficient genome editing by CRISPR-Cpf1 using CRISPR RNA with a uridinylate-rich 3′-overhang. Nat Commun. 9:3651. doi:10.1038/s41467-018-06129-w. 30194297 10.1038/s41467-018-06129-w PMC6128929

[jkab304-B4] Bindels DS , Haarbosch L, Van Weeren L, Postma M, Wiese KE, et al 2017. mScarlet: a bright monomeric red fluorescent protein for cellular imaging. Nat Methods. 14:53–56. doi:10.1038/nmeth.4074. 27869816 10.1038/nmeth.4074

[jkab304-B5] Brachmann CB , Davies A, Cost GJ, Caputo E, Li J, et al 1998. Designer deletion strains derived from *Saccharomyces cerevisiae* S288C: a useful set of strains and plasmids for PCR-mediated gene disruption and other applications. Yeast. 14:115–132. doi:10.1002/(SICI)1097-0061(19980130)14:2<115::AID-YEA204>3.0.CO;2-2. 9483801 10.1002/(SICI)1097-0061(19980130)14:2<115::AID-YEA204>3.0.CO;2-2

[jkab304-B6] Caviston JP , Longtine M, Pringle JR, Bi E. 2003. The role of Cdc42p GTPase-activating proteins in assembly of the septin ring in yeast. Mol Biol Cell. 14:4051–4066. doi:10.1091/mbc.e03-04-0247. 14517318 10.1091/mbc.E03-04-0247 PMC206999

[jkab304-B7] Concordet JP , Haeussler M. 2018. CRISPOR: intuitive guide selection for CRISPR/Cas9 genome editing experiments and screens. Nucleic Acids Res. 46:W242–W245. doi:10.1093/nar/gky354. 29762716 10.1093/nar/gky354 PMC6030908

[jkab304-B8] Chung WH , Zhu Z, Papusha A, Malkova A, Ira G. 2010. Defective resection at DNA double-strand breaks leads to *de novo* telomere formation and enhances gene targeting. PLoS Genet. 6:e1000948.doi:10.1371/journal.pgen.1000948. 20485519 10.1371/journal.pgen.1000948 PMC2869328

[jkab304-B9] Degreif D , Kremenovic M, Geiger T, Bertl A. 2018. Preloading budding yeast with all-in-one CRISPR/Cas9 vectors for easy and high-efficient genome editing. J Biol Methods. 5:e98.doi:10.14440/jbm.2018.254. 31453248 10.14440/jbm.2018.254 PMC6706142

[jkab304-B10] Deutschbauer AM , Davis RW. 2005. Quantitative trait loci mapped to single-nucleotide resolution in yeast. Nat Genet. 37:1333–1340. doi:10.1038/ng1674. 16273108 10.1038/ng1674

[jkab304-B11] DiCarlo JE , Norville JE, Mali P, Rios X, Aach J, et al 2013. Genome engineering in *Saccharomyces cerevisiae* using CRISPR-Cas systems. Nucleic Acids Res. 41:4336–4343. doi:10.1093/nar/gkt135. 23460208 10.1093/nar/gkt135 PMC3627607

[jkab304-B12] Drozdetskiy A , Cole C, Procter J, Barton GJ. 2015. JPred4: a protein secondary structure prediction server. Nucleic Acids Res. 43:W389–W394. doi:10.1093/nar/gkv332. 25883141 10.1093/nar/gkv332 PMC4489285

[jkab304-B13] Dubreuil B , Sass E, Nadav Y, Heidenreich M, Georgeson JM, et al 2019. YeastRGB: comparing the abundance and localization of yeast proteins across cells and libraries. Nucleic Acids Res. 47:D1245–D1249. doi:10.1093/nar/gky941. 30357397 10.1093/nar/gky941 PMC6324022

[jkab304-B14] Engler C , Kandzia R, Marillonnet S. 2008. A one pot, one step, precision cloning method with high throughput capability. PLoS One. 3:e3647.doi:10.1371/journal.pone.0003647. 18985154 10.1371/journal.pone.0003647 PMC2574415

[jkab304-B15] Generoso WC , Gottardi M, Oreb M, Boles E. 2016. Simplified CRISPR-Cas genome editing for *Saccharomyces cerevisiae*. J Microbiol Methods. 127:203–205. doi:10.1016/j.mimet.2016.06.020. 27327211 10.1016/j.mimet.2016.06.020

[jkab304-B16] Gietz RD , Woods RA. 2002. Transformation of yeast by lithium acetate/single-stranded carrier DNA/polyethylene glycol method. Methods Enzymol. 350:87–96. doi:10.1016/S0076-6879(02)50957-5. 12073338 10.1016/s0076-6879(02)50957-5

[jkab304-B17] Guthrie C , Fink GR. 1991. Guide to yeast genetics and molecular biology. Methods Enzymol. 194:1–933. 2005781

[jkab304-B18] Hayashi A , Tanaka K. 2019. Short-homology-mediated CRISPR/Cas9-based method for genome editing in fission yeast. G3 (Bethesda). 9:1153–1163. doi:10.1534/g3.118.200976. 30755408 10.1534/g3.118.200976 PMC6469419

[jkab304-B19] Hickman MJ , Gibney PA, McClean MN, McIsaac RS, Macinskas J, et al 2011. Fast-acting and nearly gratuitous induction of gene expression and protein depletion in *Saccharomyces cerevisiae*. Mol Biol Cell. 22:4447–4459. doi:10.1091/mbc.e11-05-0466. 21965290 10.1091/mbc.E11-05-0466 PMC3216669

[jkab304-B20] Hieter P , Pridmore D, Hegemann JH, Thomas M, Davis RW, et al 1985. Functional selection and analysis of yeast centromeric DNA. Cell. 42:913–921. doi:10.1016/0092-8674(85)90287-9. 2996783 10.1016/0092-8674(85)90287-9

[jkab304-B21] Hsu PD , Lander ES, Zhang F. 2014. Development and applications of CRISPR-Cas9 for genome engineering. Cell. 157:1262–1278. doi:10.1016/j.cell.2014.05.010. 24906146 10.1016/j.cell.2014.05.010 PMC4343198

[jkab304-B22] Huh WK , Falvo JV, Gerke LC, Carroll AS, Howson RW, et al 2003. Global analysis of protein localization in budding yeast. Nature. 425:686–691. doi:10.1038/nature02026. 14562095 10.1038/nature02026

[jkab304-B23] Jessop-Fabre MM , Jakočiūnas T, Stovicek V, Dai Z, Jensen MK, et al 2016. EasyClone-MarkerFree: a vector toolkit for marker-less integration of genes into *Saccharomyces cerevisiae* via CRISPR-Cas9. Biotechnol J. 11:1110–1117. doi:10.1002/biot.201600147. 27166612 10.1002/biot.201600147 PMC5094547

[jkab304-B24] Jinek M , Chylinski K, Fonfara I, Hauer M, Doudna JA, et al 2012. A programmable dual-RNA-guided DNA endonuclease in adaptive bacterial immunity. Science. 337:816–821. doi:10.1126/science.1225829. 22745249 10.1126/science.1225829 PMC6286148

[jkab304-B25] Kim D , Kim JSJ, Hur JK, Been KW, Yoon SH, et al 2016. Genome-wide analysis reveals specificities of Cpf1 endonucleases in human cells. Nat Biotechnol. 34:863–868. doi:10.1038/nbt.3609. 27272384 10.1038/nbt.3609

[jkab304-B26] Kleinstiver BP , Sousa AA, Walton RT, Tak YE, Hsu JY, et al 2019. Engineered CRISPR-Cas12a variants with increased activities and improved targeting ranges for gene, epigenetic and base editing. Nat Biotechnol. 37:276–282. doi:10.1038/s41587-018-0011-0. 30742127 10.1038/s41587-018-0011-0 PMC6401248

[jkab304-B27] Kleinstiver BP , Tsai SQ, Prew MS, Nguyen NT, Welch MM, et al 2016. Genome-wide specificities of CRISPR-Cas Cpf1 nucleases in human cells. Nat Biotechnol. 34:869–874. doi:10.1038/nbt.3620. 27347757 10.1038/nbt.3620 PMC4980201

[jkab304-B28] Koshland D , Kent JC, Hartwell LH. 1985. Genetic analysis of the mitotic transmission of minichromosomes. Cell. 40:393–403. doi:10.1016/0092-8674(85)90153-9. 3881185 10.1016/0092-8674(85)90153-9

[jkab304-B29] Laughery MF , Hunter T, Brown A, Hoopes J, Ostbye T, et al 2015. New vectors for simple and streamlined CRISPR-Cas9 genome editing in *Saccharomyces cerevisiae*. Yeast. 32:711–720. doi:10.1002/yea.3098. 26305040 10.1002/yea.3098 PMC4715497

[jkab304-B30] Li JJ , Herskowitz I. 1993. Isolation of *ORC6*, a component of the yeast origin recognition complex by a one-hybrid system. Science. 262:1870–1874. doi:10.1126/science.8266075. 8266075 10.1126/science.8266075

[jkab304-B31] Longtine MS , McKenzie A, Demarini DJ, Shah NG, Wach A, et al 1998. Additional modules for versatile and economical PCR-based gene deletion and modification in *Saccharomyces cerevisiae*. Yeast. 14:953–961. doi:10.1002/(SICI)1097-0061(199807)14:10<953::AID-YEA293>3.0.CO;2-U. 9717241 10.1002/(SICI)1097-0061(199807)14:10<953::AID-YEA293>3.0.CO;2-U

[jkab304-B32] McKenzie KQ , Jones EW. 1977. Mutants of formyltetrahydrofolate interconversion pathway of *Saccharomyces cerevisiae*. Genetics. 86:85–102. 328341 10.1093/genetics/86.1.85 PMC1213674

[jkab304-B33] Naito Y , Hino K, Bono H, Ui-Tei K. 2015. CRISPRdirect: software for designing CRISPR/Cas guide RNA with reduced off-target sites. Bioinformatics. 31:1120–1123. doi:10.1093/bioinformatics/btu743. 25414360 10.1093/bioinformatics/btu743 PMC4382898

[jkab304-B34] Ran FA , Cong L, Yan WX, Scott DA, Gootenberg JS, et al 2015. *In vivo* genome editing using *Staphylococcus aureus* Cas9. Nature. 520:186–191. doi:10.1038/nature14299. 25830891 10.1038/nature14299 PMC4393360

[jkab304-B35] Richardson CD , Li JJ. 2014. Regulatory mechanisms that prevent re-initiation of DNA replication can be locally modulated at origins by nearby sequence elements. PLoS Genet. 10:e1004358.doi:10.1371/journal.pgen.1004358. 24945837 10.1371/journal.pgen.1004358 PMC4063666

[jkab304-B36] Schindelin J , Arganda-Carreras I, Frise E, Kaynig V, Longair M, et al 2012. Fiji: an open-source platform for biological-image analysis. Nat Methods. 9:676–682. doi:10.1038/nmeth.2019. 22743772 10.1038/nmeth.2019 PMC3855844

[jkab304-B37] Schulz VP , Zakian VA. 1994. The *Saccharomyces PIF1* DNA helicase inhibits telomere elongation and *de novo* telomere formation. Cell. 76:145–155. doi:10.1016/0092-8674(94)90179-1. 8287473 10.1016/0092-8674(94)90179-1

[jkab304-B38] Shaner NC , Lambert GG, Chammas A, Ni Y, Cranfill PJ, et al 2013. A bright monomeric green fluorescent protein derived from *Branchiostoma lanceolatum*. Nat Methods. 10:407–409. doi:10.1038/nmeth.2413 23524392 10.1038/nmeth.2413 PMC3811051

[jkab304-B39] Sternberg SH , Redding S, Jinek M, Greene EC, Doudna JA. 2014. DNA interrogation by the CRISPR RNA-guided endonuclease Cas9. Nature. 507:62–67. doi:10.1038/nature13011. 24476820 10.1038/nature13011 PMC4106473

[jkab304-B40] Stoler S , Keith KC, Curnick KE, Fitzgerald-Hayes M. 1995. A mutation in *CSE4*, an essential gene encoding a novel chromatin-associated protein in yeast, causes chromosome nondisjunction and cell cycle arrest at mitosis. Genes Dev. 9:573–586. doi:10.1101/gad.9.5.573. 7698647 10.1101/gad.9.5.573

[jkab304-B41] Świat MA , Dashko S, Den Ridder M, Wijsman M, Van Der Oost J, et al. 2017. FnCpf1: a novel and efficient genome editing tool for Saccharomyces cerevisiae. Nucleic Acids Res. 45:12585–12598. 10.1093/nar/gkx1007 29106617 29106617 PMC5716609

[jkab304-B42] Tycko J , Barrera LA, Huston NC, Friedland AE, Wu X, et al 2018. Pairwise library screen systematically interrogates *Staphylococcus aureus* Cas9 specificity in human cells. Nat Commun. 9:2962. doi:10.1038/s41467-018-05391-2. 30054474 10.1038/s41467-018-05391-2 PMC6063963

[jkab304-B43] Verwaal R , Buiting-Wiessenhaan N, Dalhuijsen S, Roubos JA. 2018. CRISPR/Cpf1 enables fast and simple genome editing of *Saccharomyces cerevisiae*. Yeast. 35:201–211. doi:10.1002/yea.3278. 28886218 10.1002/yea.3278 PMC5836994

[jkab304-B44] Wisniewski J , Hajj B, Chen J, Mizuguchi G, Xiao H, et al 2014. Imaging the fate of histone Cse4 reveals *de novo* replacement in S phase and subsequent stable residence at centromeres. Elife. 3:e02203. doi:10.7554/eLife.02203. 24844245 10.7554/eLife.02203 PMC4067749

[jkab304-B45] Yan K , Yang J, Zhang Z, McLaughlin SH, Chang L, et al 2019. Structure of the inner kinetochore CCAN complex assembled onto a centromeric nucleosome. Nature. 574:278–282. doi:10.1038/s41586-019-1609-1. 31578520 10.1038/s41586-019-1609-1 PMC6859074

[jkab304-B46] Zetsche B , Gootenberg JS, Abudayyeh OO, Slaymaker IM, Makarova KS, et al 2015. Cpf1 is a single RNA-guided endonuclease of a class 2 CRISPR-Cas system. Cell. 163:759–771. doi:10.1016/j.cell.2015.09.038. 26422227 10.1016/j.cell.2015.09.038 PMC4638220

[jkab304-B47] Zhang Y , Wang J, Wang Z, Zhang Y, Shi S, et al 2019. A gRNA-tRNA array for CRISPR-Cas9 based rapid multiplexed genome editing in *Saccharomyces cerevisiae*. Nat Commun. 10:1053. doi:10.1038/s41467-019-09005-3. 30837474 10.1038/s41467-019-09005-3 PMC6400946

[jkab304-B48] Zheng T , Hou Y, Zhang P, Zhang Z, Xu Y, et al 2017. Profiling single-guide RNA specificity reveals a mismatch sensitive core sequence. Sci Rep. 7:40638.doi:10.1038/srep40638. 28098181 10.1038/srep40638 PMC5241822

[jkab304-B49] Zhou Z , Feng H, Zhou BR, Ghirlando R, Hu K, et al 2011. Structural basis for recognition of centromere histone variant CenH3 by the chaperone Scm3. Nature. 472:234–238. doi:10.1038/nature09854. 21412236 10.1038/nature09854 PMC3077455

