# Supplementary material for: Simple-to-use CRISPR-SpCas9/SaCas9/AsCas12a vector series for genome editing in Saccharomyces cerevisiae
Source: G3 (Bethesda). 2021 Aug 30;11(12):jkab304. doi: 10.1093/g3journal/jkab304 (PMC8664446; doi:10.1093/g3journal/jkab304)
Supplement: jkab304_Supplementary_Figures [file jkab304_supplementary_figures.pdf]

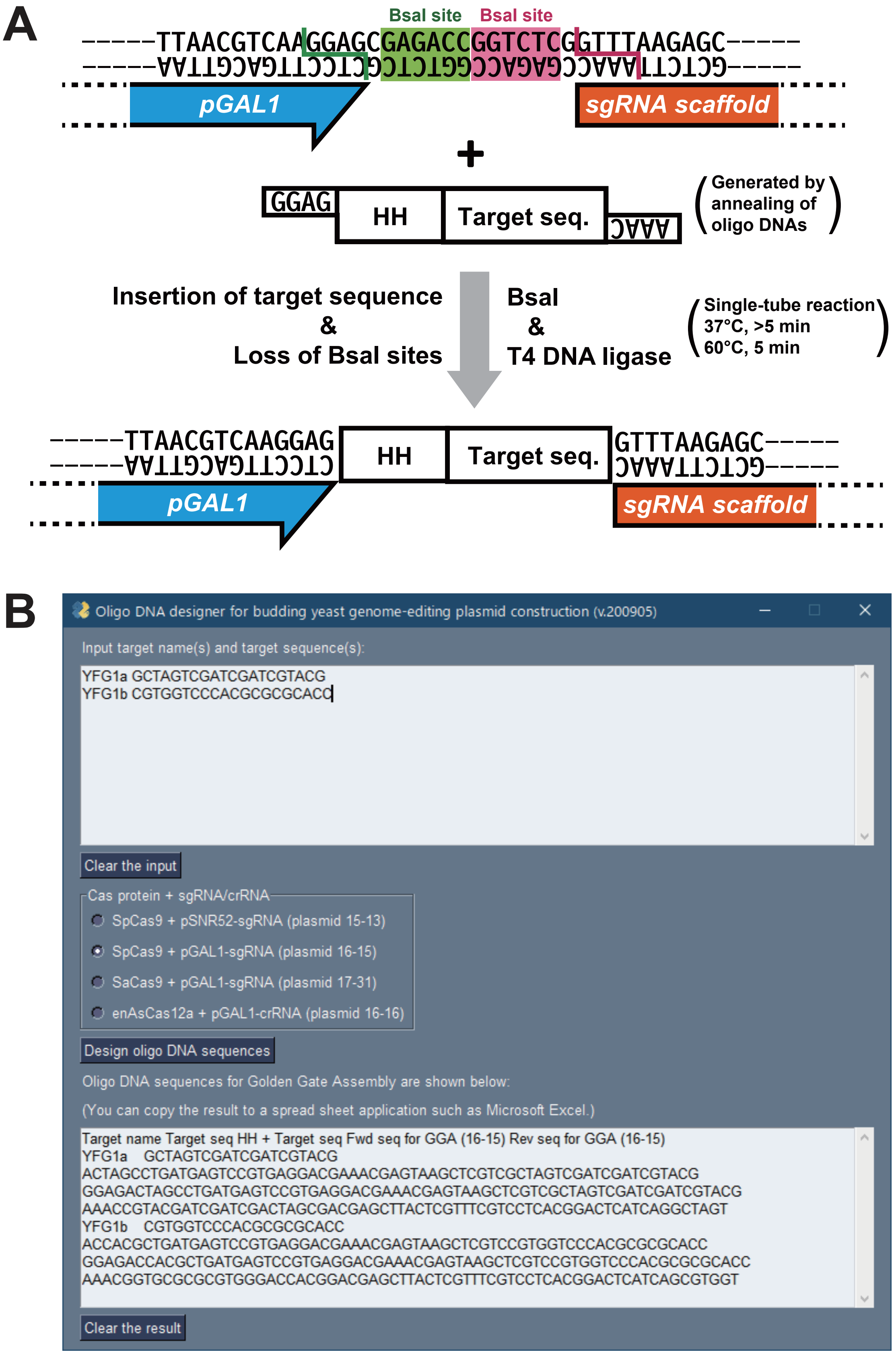

Figure S1. Golden Gate Assembly of genome-editing plasmids and a program to design ODN sequences. (A) Process to construct a genome-editing plasmid by Golden Gate Assembly. This panel shows an example for SpCas9 + pGAL1-sgRNA system. HH, hammerhead ribozyme-encoding sequence. (B) Screenshot of the program to automatically design the ODN sequences for Golden Gate Assembly. Upon inputting target sequences with their names to the box at the top, followed by selecting a backbone vector at the middle part, ODN sequences are displayed in the box at the bottom.

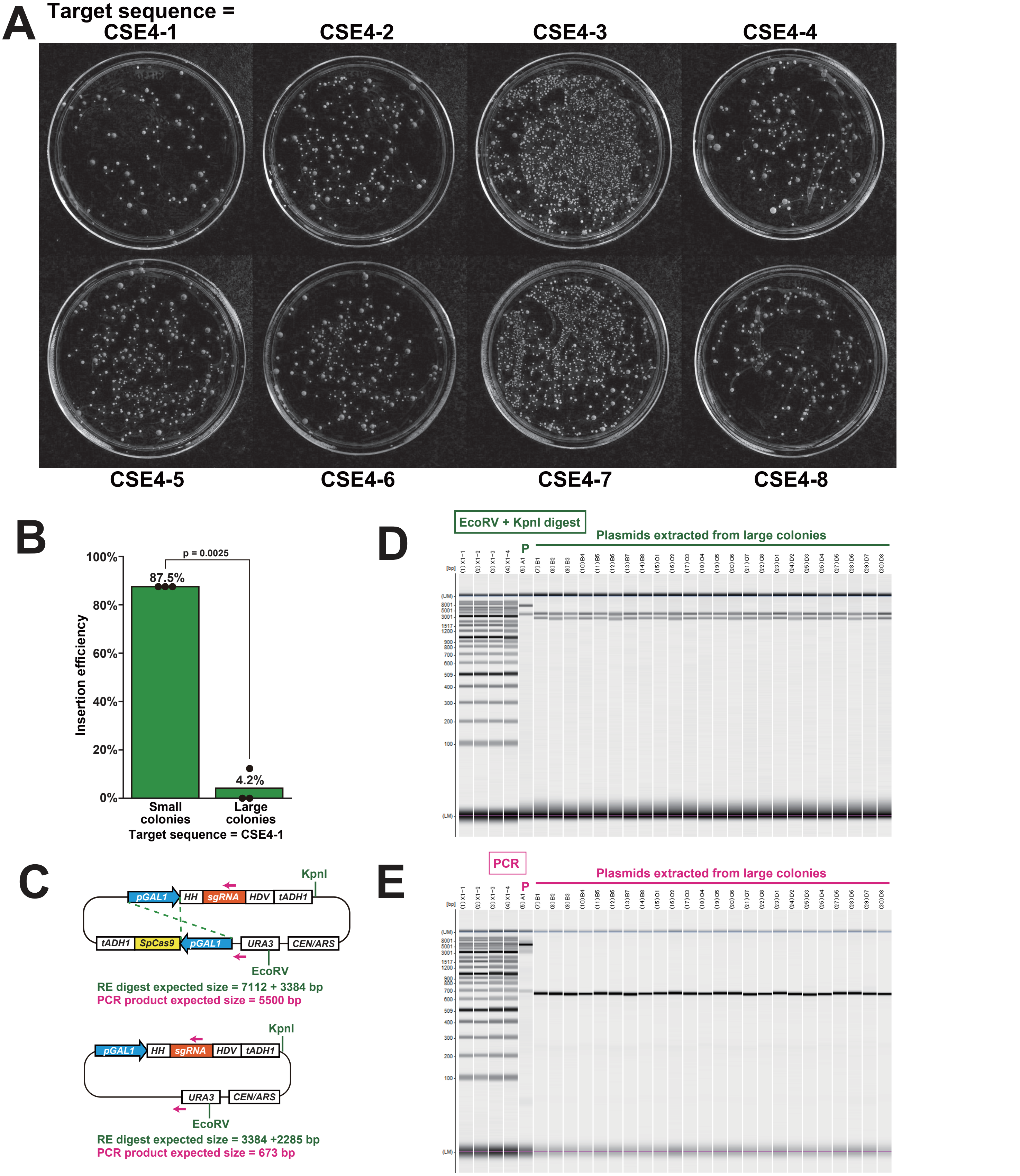

Figure S2. Characterization of large and small colonies obtained by transformation of genome-editing plasmids.
(A) Representative images of the colonies on galactose-containing agar plates. The cells were transformed with the pGAL1-SpCas9 + pGAL1-sgRNA plasmid for insertion of the mNeonGreen-encoding gene to *CSE4* (Figures 3B and 3C). Each plate is labeled with the target sequence used in Figure 3B. (B) Insertion efficiency at the CSE4-1 target sequence. Green bars indicate the insertion efficiency (n = 8 for each of 3 biological replicates) of small colonies (left) and large colonies (right). The p-value of paired-samples t-test is shown. (C) Structures of the parental plasmid and its putative derivative generated via intramolecular recombination between the two *GAL1* promoters. Restriction enzyme sites used for plasmid DNA digestion are indicated in green. Magenta arrows indicate the positions of primers used for PCR. (D) Plasmid DNAs digested with EcoRV and KpnI analyzed by a microchip electrophoresis system. P, parental plasmid. (E) PCR products analyzed by a microchip electrophoresis system. P, parental plasmid.

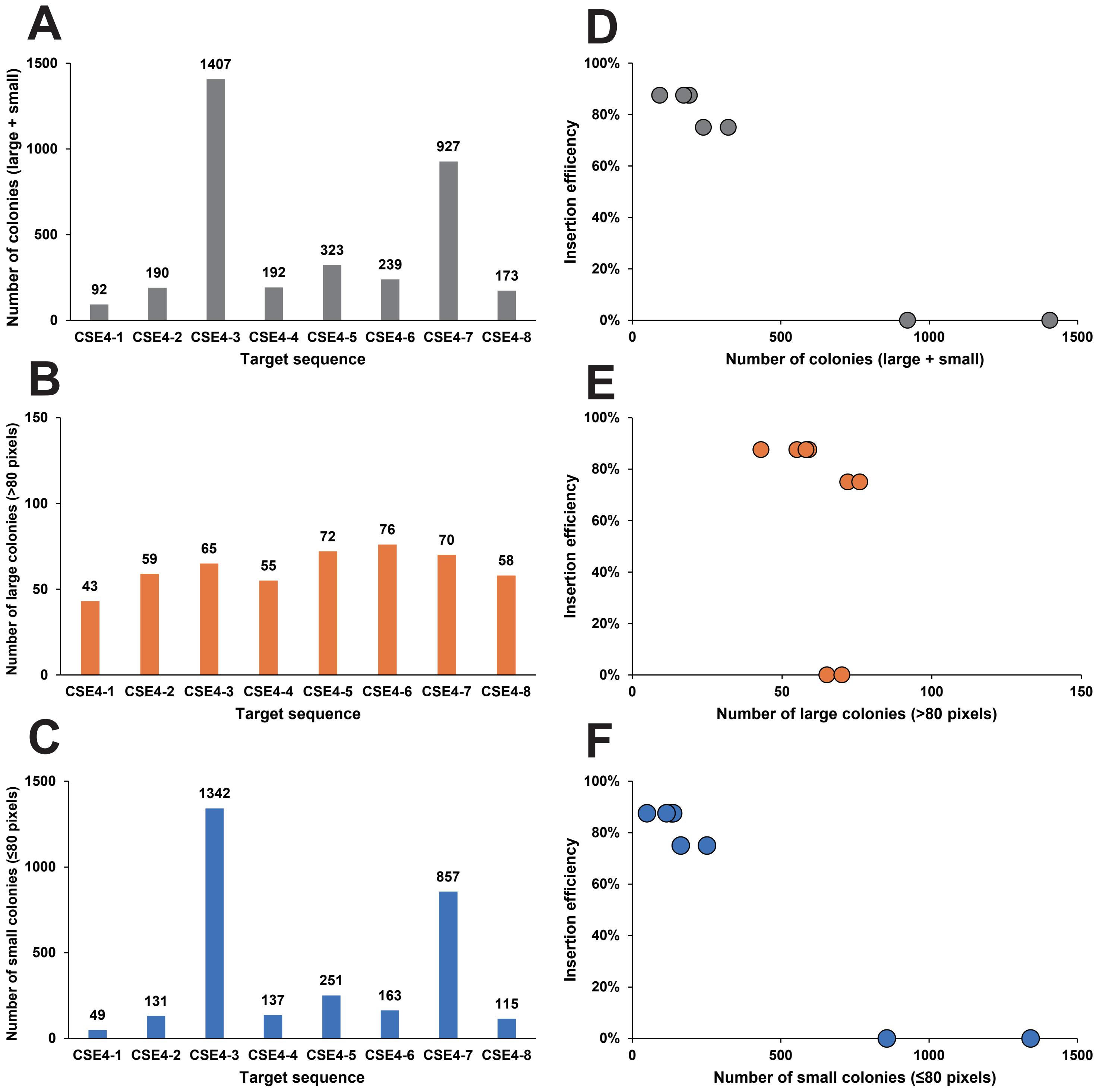

Figure S3. Numbers of large and small colonies and insertion efficiency.
(A, B, C) Number of total colonies (A), large colonies (B), and small colonies (C) on the plates shown in Figure S2A. (D, E, F) Relationship between number of colonies and the efficiency of mNeonGreen gene fragment insertion.

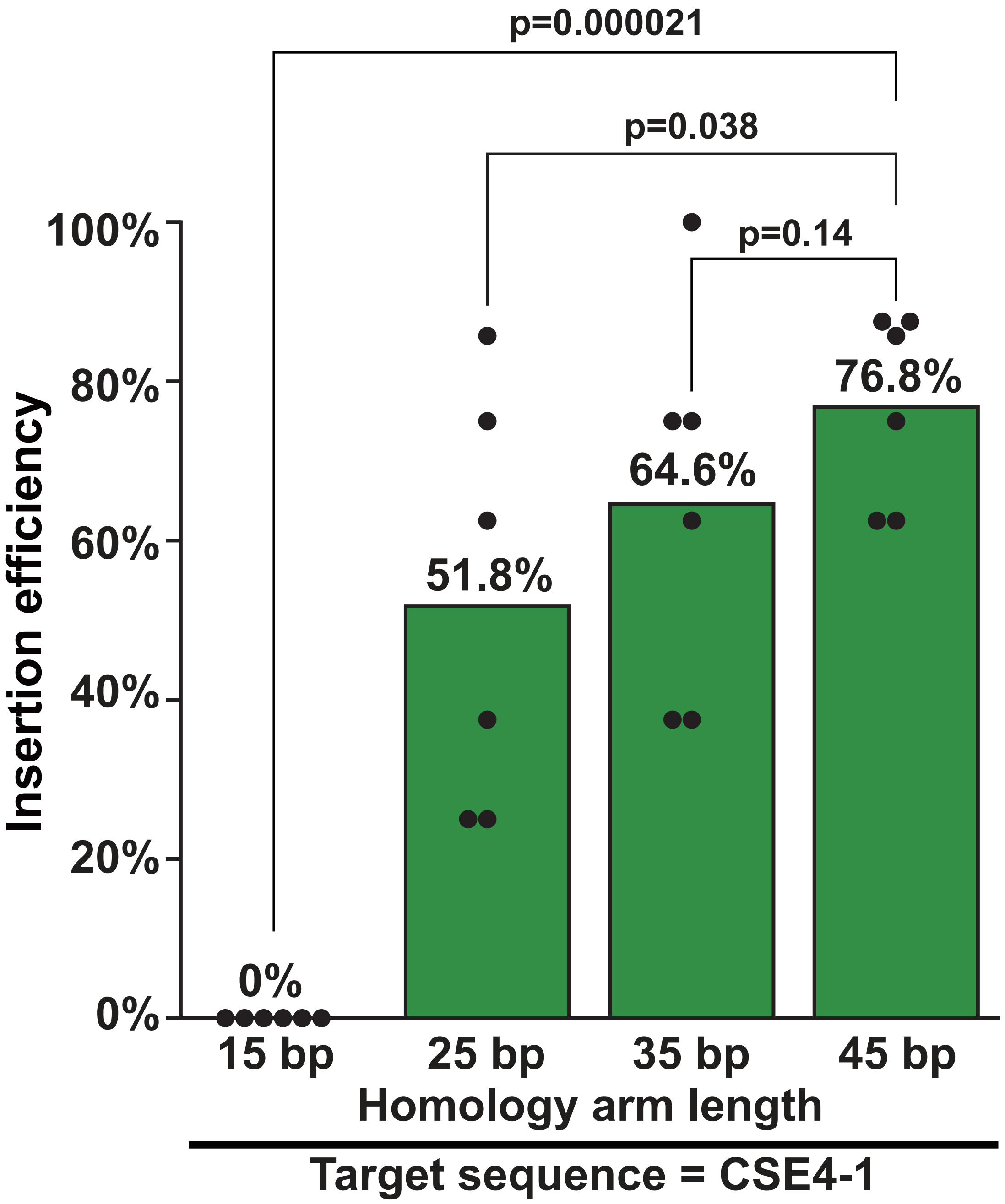

Figure S4. Effects of homology arm length on gene fragment insertion.
Insertion efficiency at the CSE4-1 target sequence is indicated for donor PCR fragments harboring homology arms of four different lengths. Green bars indicate the insertion efficiency (n = 8 for each of 6 biological replicates). Only small colonies were examined.

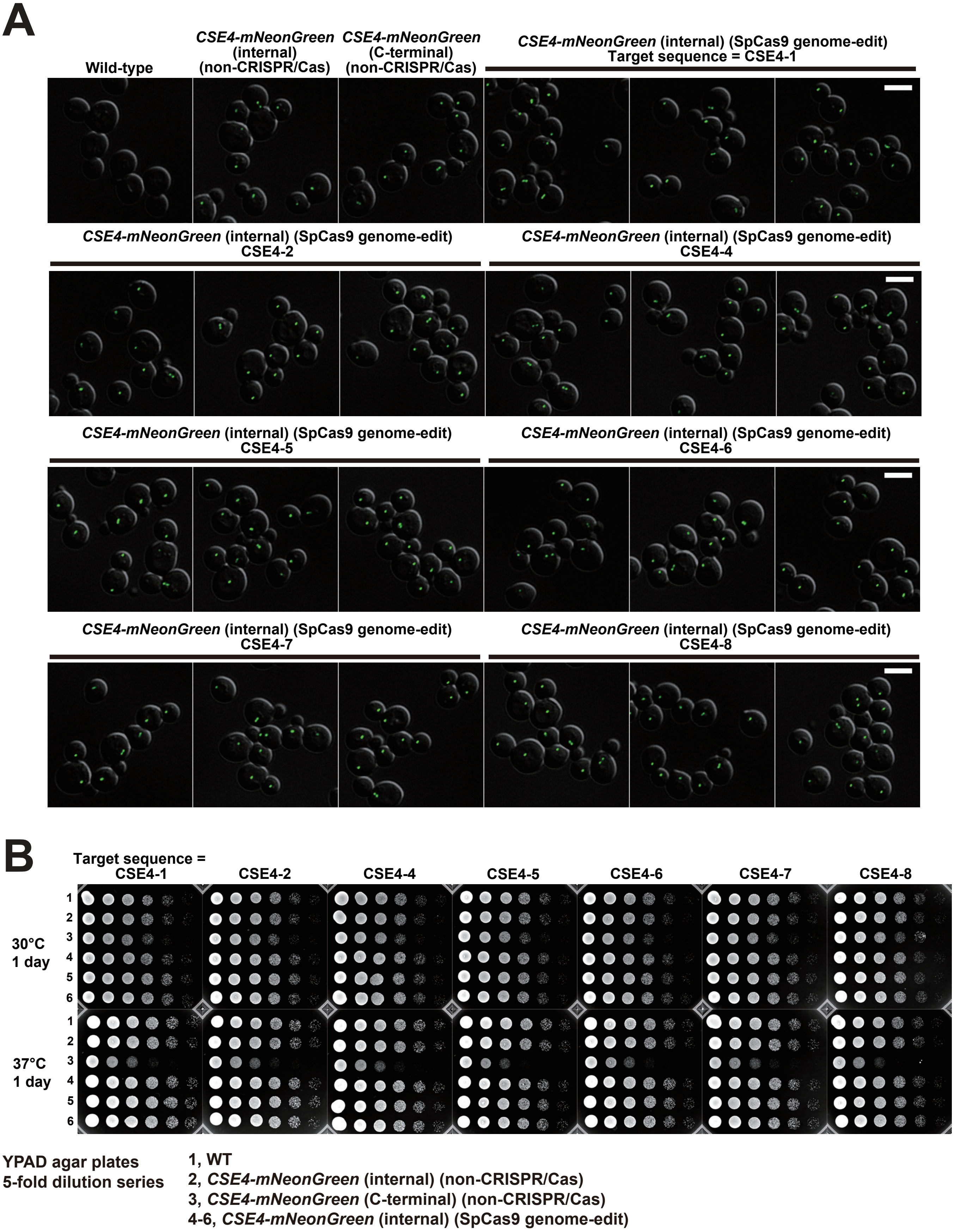

Figure S5. Characterization of *CSE4-mNeonGreen* cells generated by SpCas9-mediated genome editing.
(A) Representative images of the wild-type cells, the *CSE4-mNeonGreen* cells generated by the conventional plasmid integration method, and the genome-edited *CSE4-mNeonGreen* cells. Images are composed by the superimposition of DIC images (grayscale) and mNeonGreen fluorescent images (green). The target sequence names are shown above the images. Scale bar, 5 µm. (B) Images of cells grown on YPAD plates at 30°C and 37°C for a day. Overnight culture in YPAD liquid medium of each strain was diluted to the same cell density among the samples, serially diluted (5-fold), and spotted on YPAD agar plates (5 µL per spot).

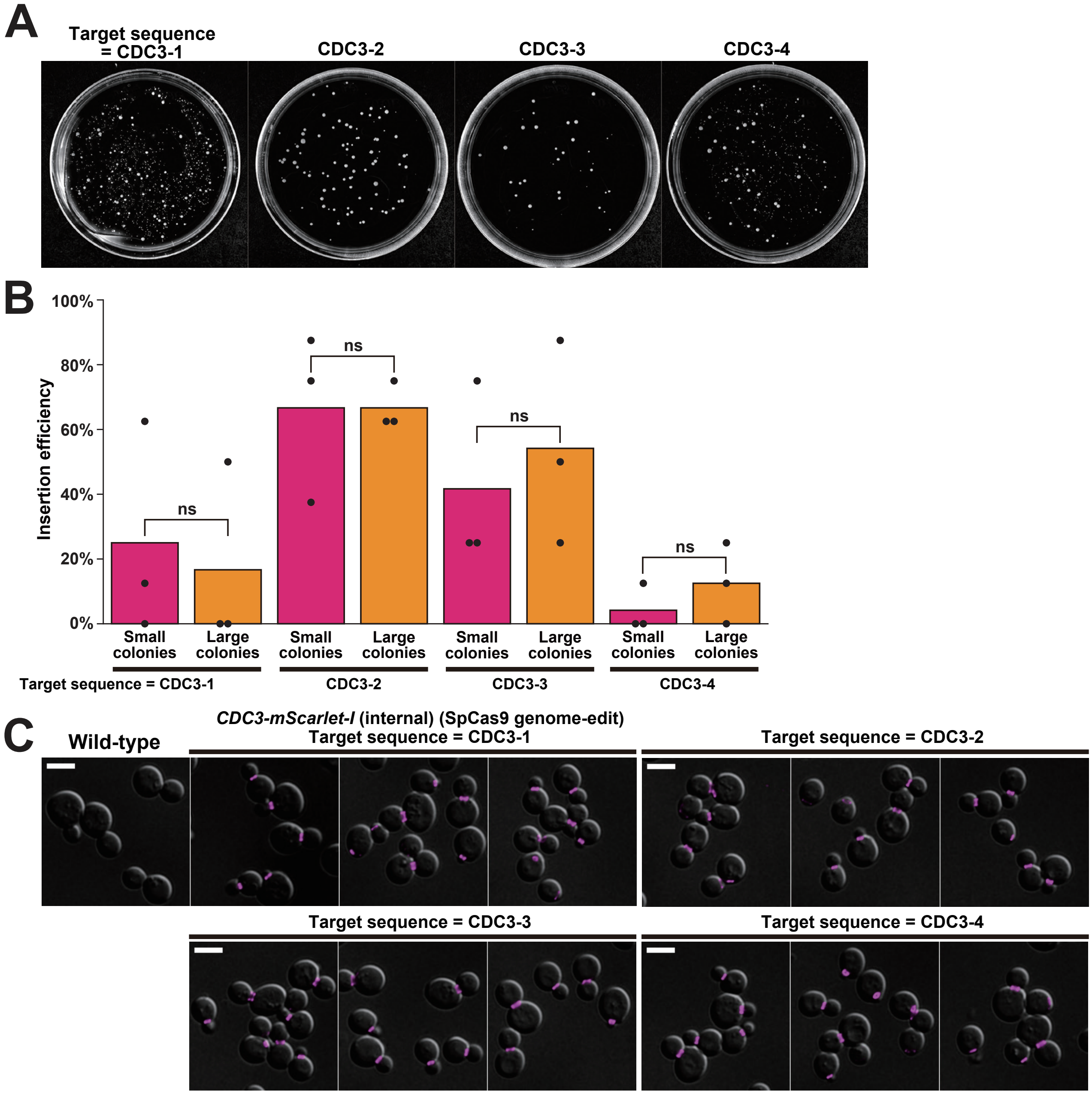

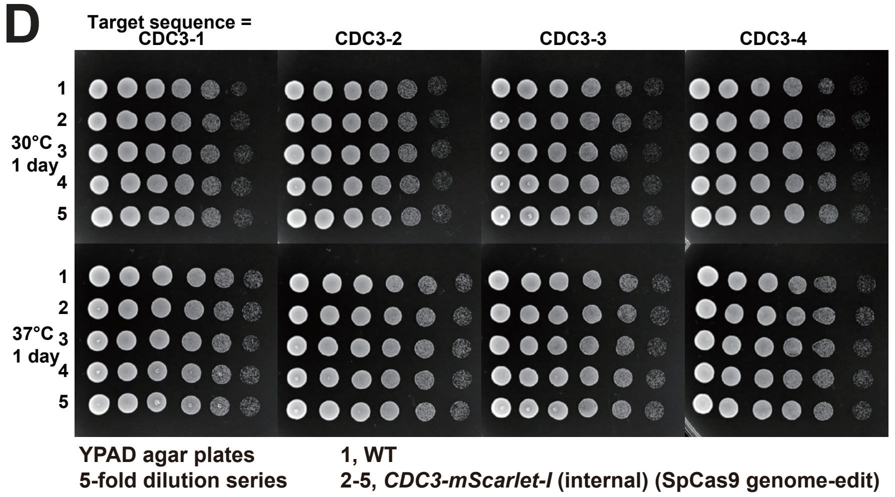

Figure S6. Characterization of *CDC3-mScarlet-I* cells generated by SpCas9-mediated genome editing. (A) Representative images of the colonies on galactose-containing agar plates. The cells were transformed with the pGAL1-SpCas9 + pSNR52-sgRNA plasmid. The target sequence names used for genome editing are shown above the plates. (B) Insertion efficiency at the four different target sequences in the *CDC3* gene. Magenta and orange bars indicate the insertion efficiency of small and large colonies, respectively (n = 8 for each of 3 biological replicates). For each target sequence, insertion efficiency does not show a statistically significant difference between small and large colonies (paired-samples t-test). (C) Representative images of the wild-type cells and the genome-edited *CDC3-mScarlet-I* cells. Images are composed by the superimposition of DIC images (grayscale) and mScarlet-I fluorescent images (magenta). The target sequence names are shown above the images. Scale bar, 5 µm. (D) Images of cells grown on YPAD plates at 30°C and 37°C for a day. Overnight culture in YPAD liquid medium of each strain was diluted to the same cell density among the samples, serially diluted (5-fold), and spotted on YPAD agar plates (5 µL per spot).

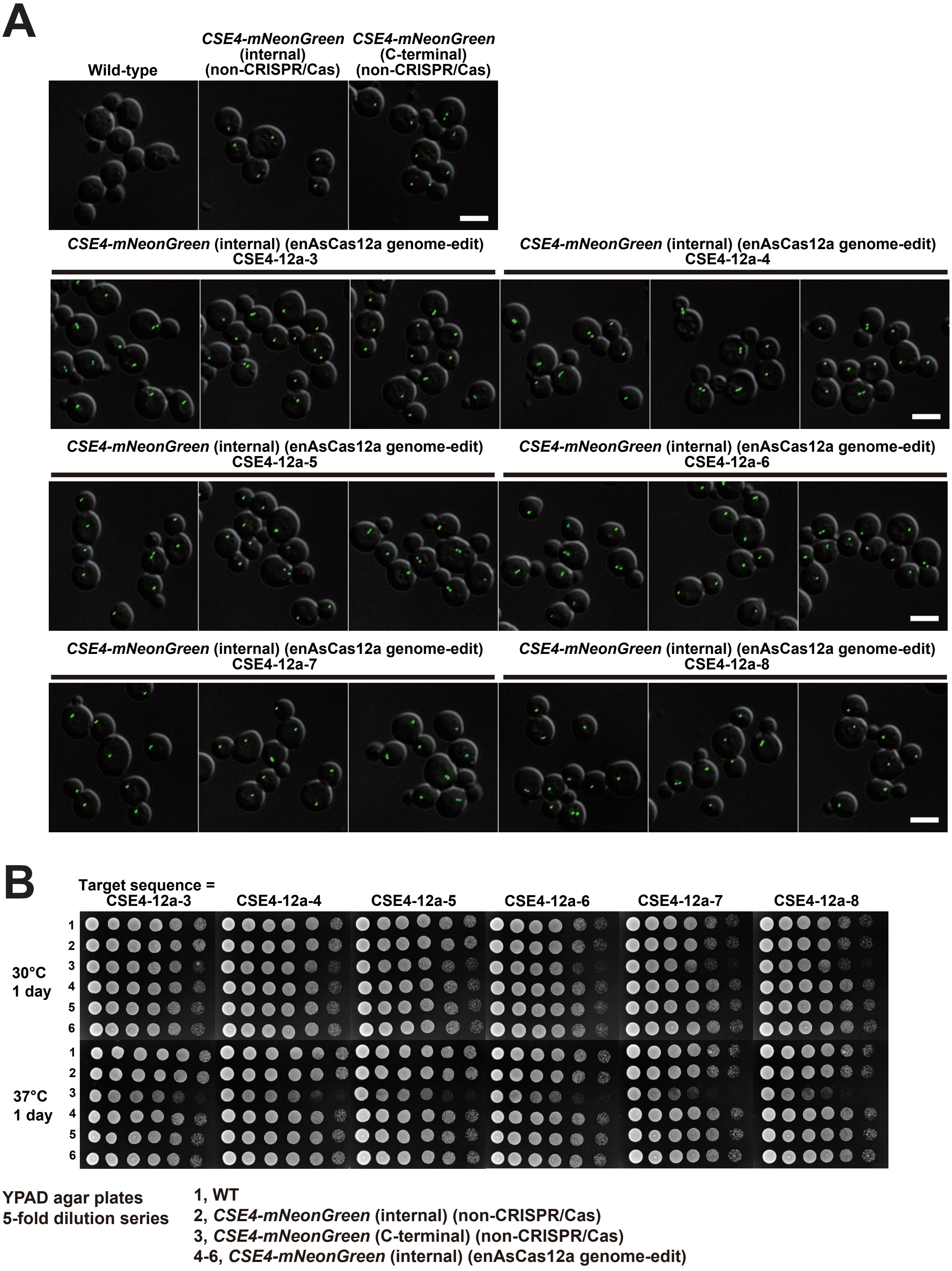

Figure S7. Characterization of *CSE4-mNeonGreen* cells generated by enAsCas12a-mediated genome editing. (A) Representative images of the wild-type cells, the *CSE4-mNeonGreen* cells generated by the conventional plasmid integration method, and the genome-edited *CSE4-mNeonGreen* cells. Images are composed by the superimposition of DIC images (grayscale) and mNeonGreen fluorescent images (green). The target sequence names are shown above the images. Scale bar, 5 µm. (B) Images of cells grown on YPAD plates at 30°C and 37°C for a day. Overnight culture in YPAD liquid medium of each strain was diluted to the same cell density among the samples, serially diluted (5-fold), and spotted on YPAD agar plates (5 µL per spot).

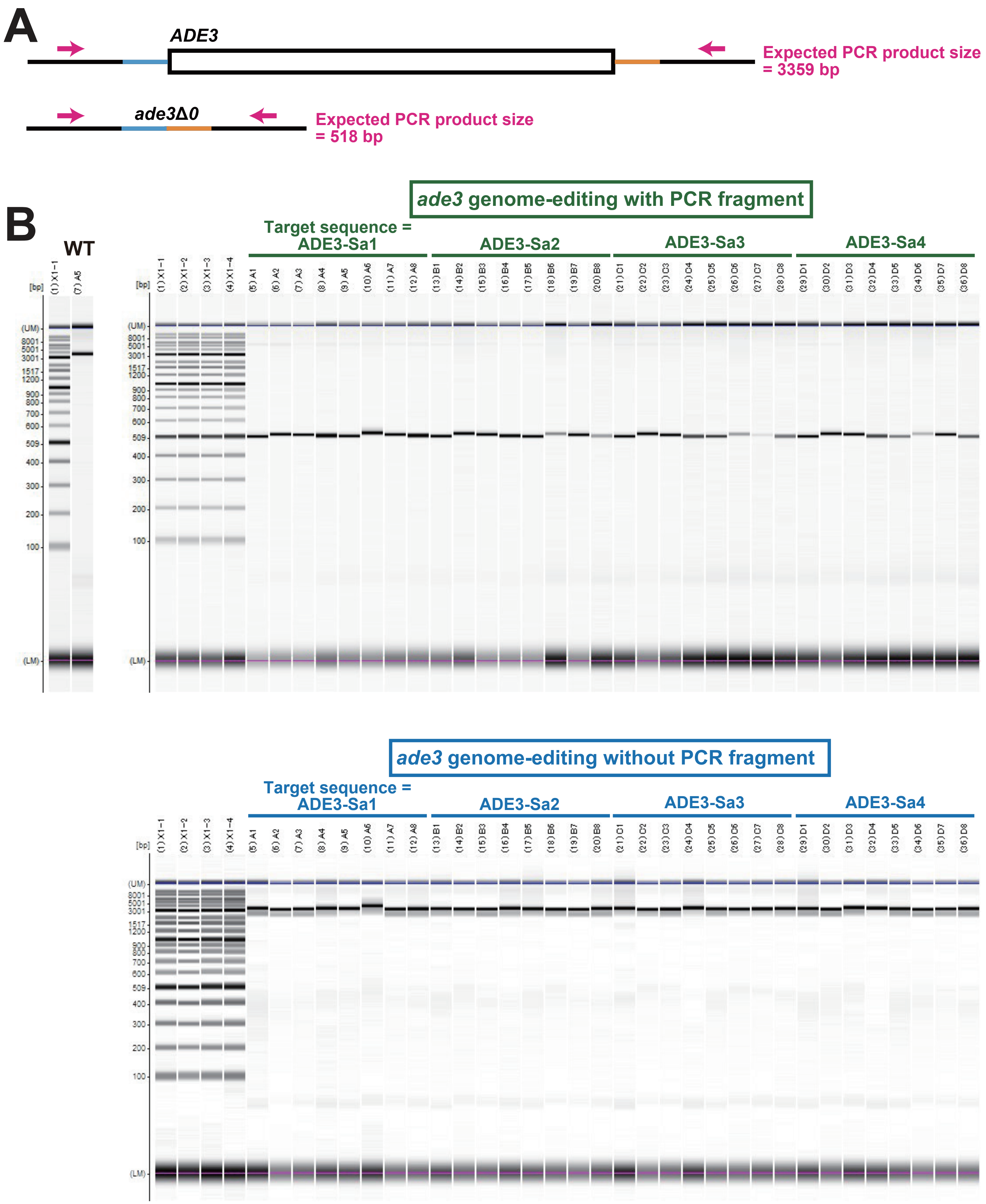

Figure S8. PCR characterization of white colonies obtained by SaCas9-mediated genome editing of *ADE3*. (A) Schematic representation of the *ADE3* locus (before genome editing) and the *ade3*Δ0 locus (after genome editing). Magenta arrows indicate the primer positions used for PCR check. (B) PCR products from white colonies appeared on adenine-limited galactose-containing agar plates. The transformation was performed with (top) and without (bottom) the donor PCR fragment shown in Figure 4.

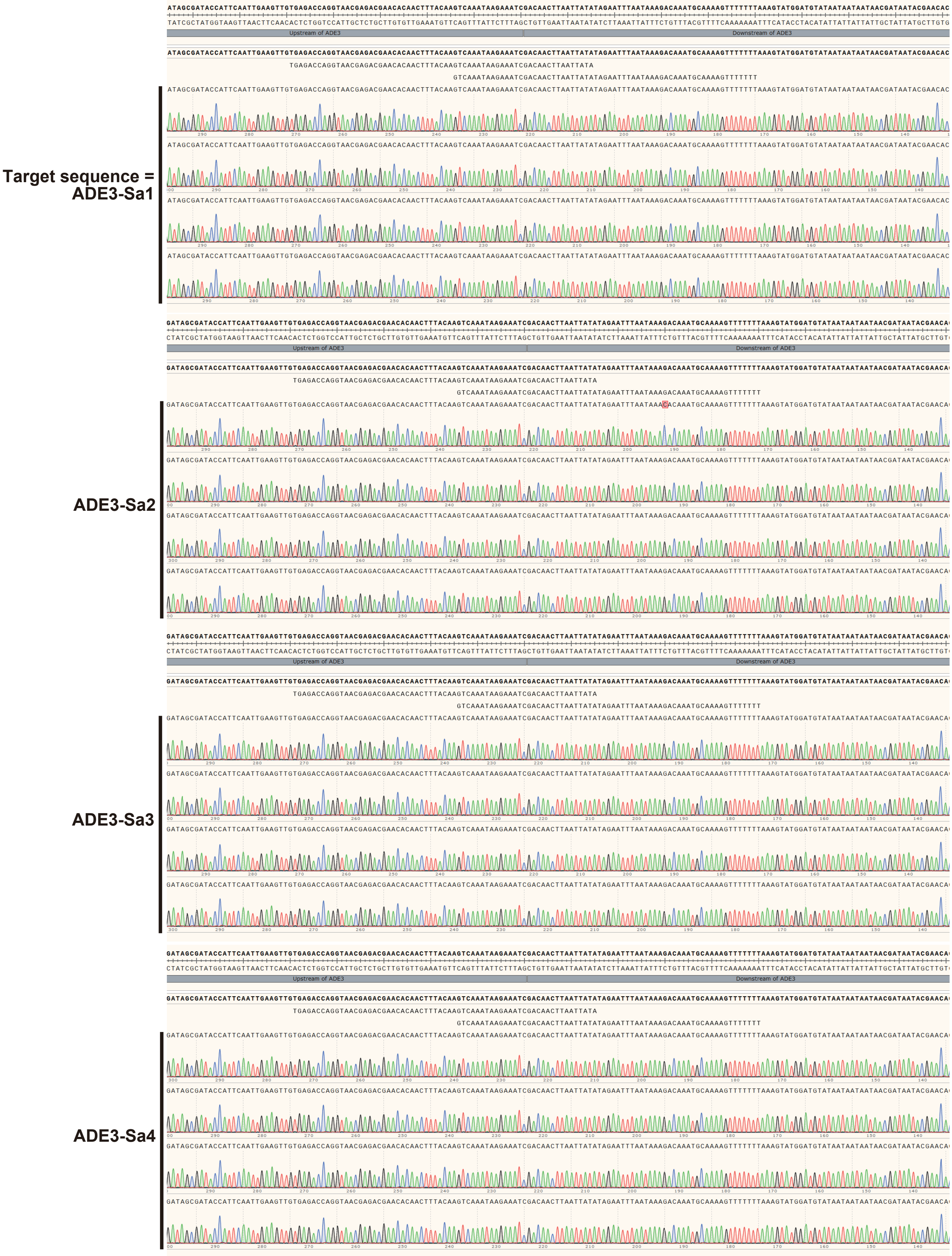

Figure S9. Nucleotide sequences of *ade3*Δ0 alleles generated by SaCas9-mediated genome editing with a donor PCR fragment.

Nucleotide sequences are shown for the *ade3*Δ0 alleles generated by transformation of the individual genome-editing plasmids with the donor PCR fragment (Figure 4A). The expected *ade3*Δ0 sequence is shown at the top. Two horizontal gray bars are the upstream and downstream sequences of the *ADE3* gene.

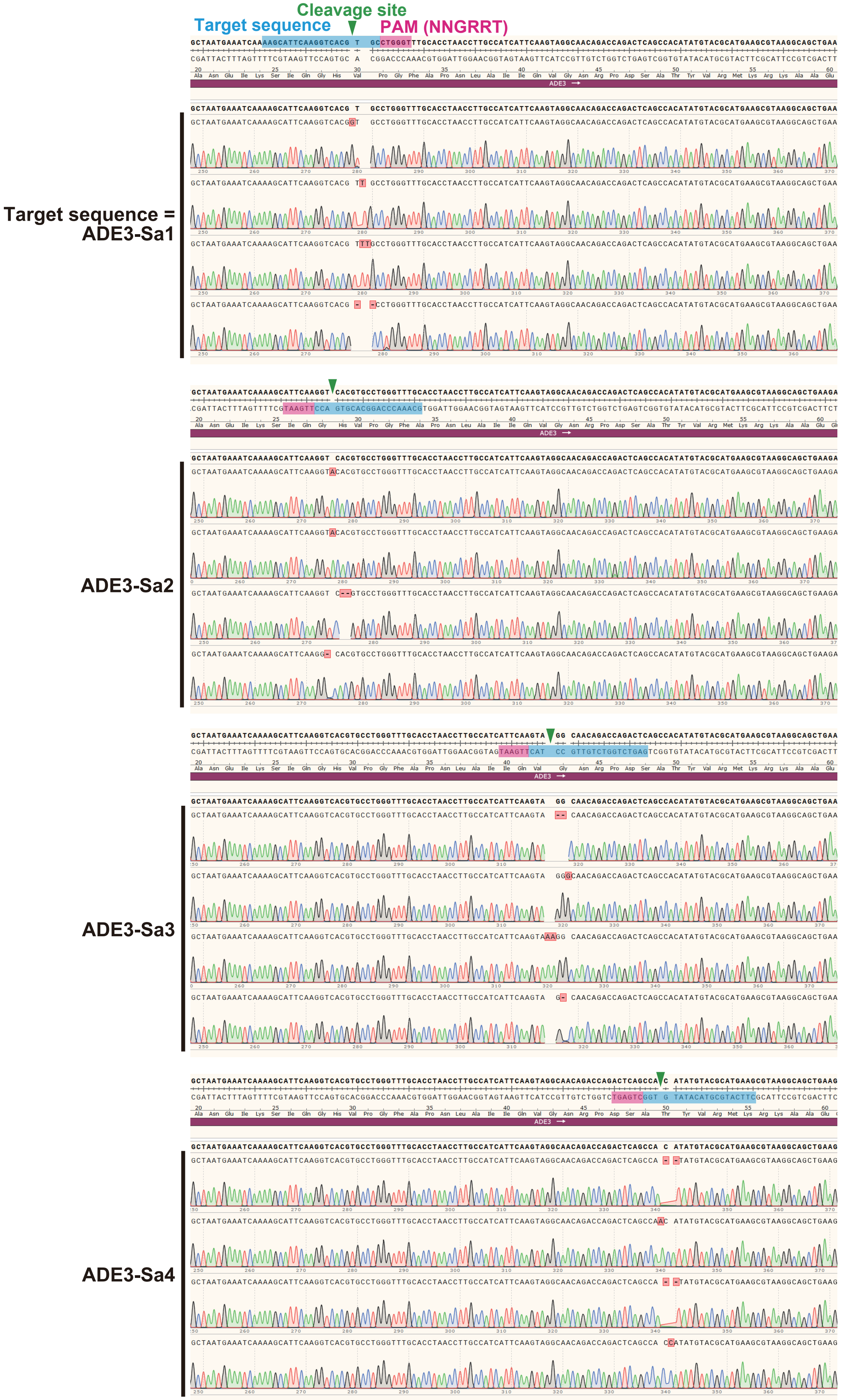

Figure S10. Nucleotide sequences of *ade3* alleles generated by genome editing without a donor PCR fragment. Nucleotide sequences are shown for the *ade3* alleles generated by transformation of the individual genome-editing plasmids without the donor PCR fragment (Figure 4A). At the top of each panel, the unedited *ADE3* sequence is shown. The target sequence and the PAM are highlighted with blue and magenta, respectively. Green triangles indicate the expected cleavage site by SaCas9. Inserted or deleted nucleotides are boxed by red and highlighted with pink in each sequence trace.

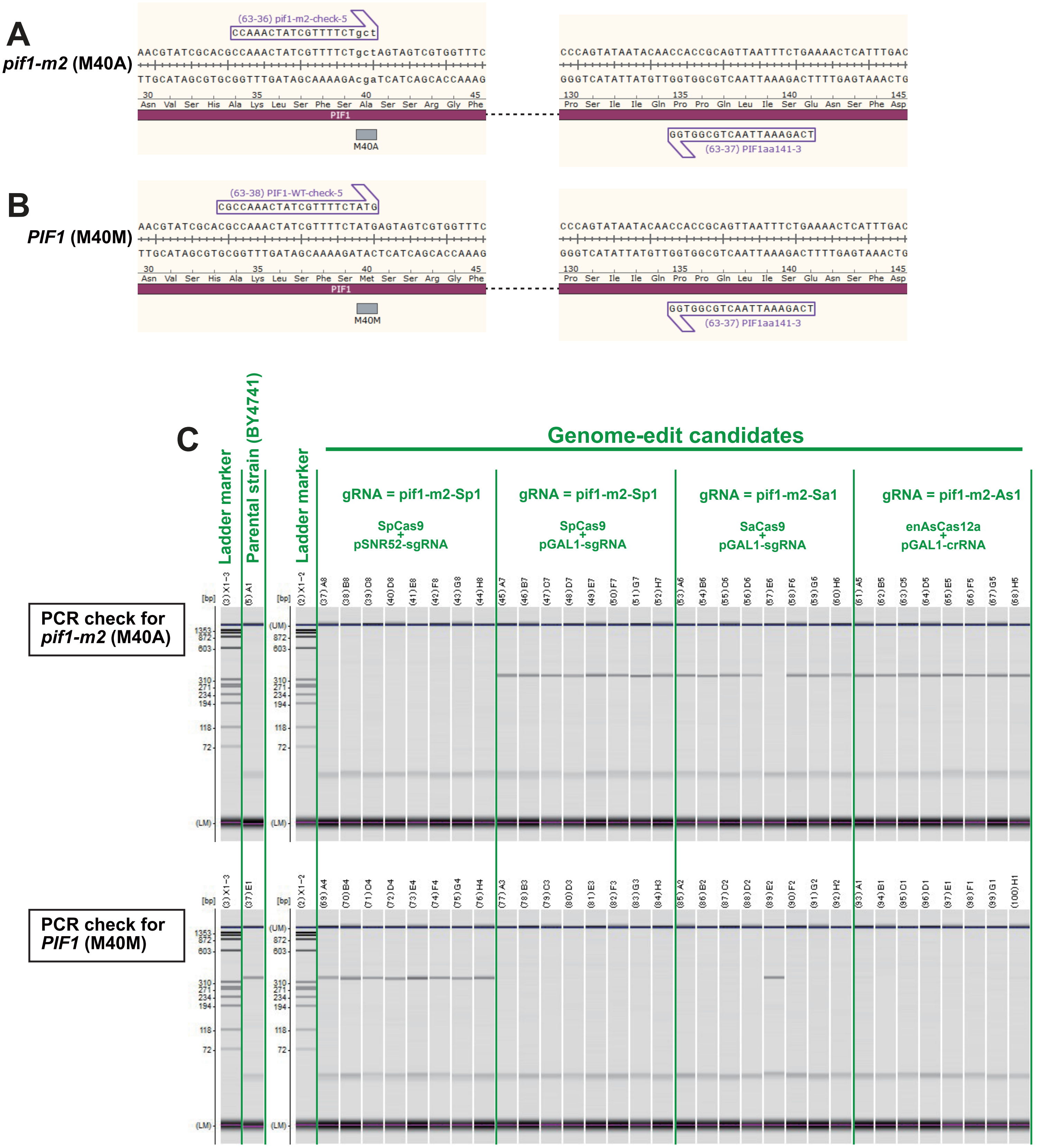

Figure S11. PCR examination of *pif1-m2* (M40A) allele.

(A, B) Primers for PCR assay to distinguish the *pif1-m2* (M40A) allele and the wild-type *PIF1* (M40M) allele. (C) An example of results of PCR assay to distinguish the *pif1-m2* (M40A) allele and the wild-type *PIF1* (M40M) allele applied to candidate genome-edited clones. Top, PCR check for the *pif1-m2* (M40A) allele. Bottom, PCR check for the wild-type *PIF1* (M40M) allele. Each clone was examined using the two pairs of primers.

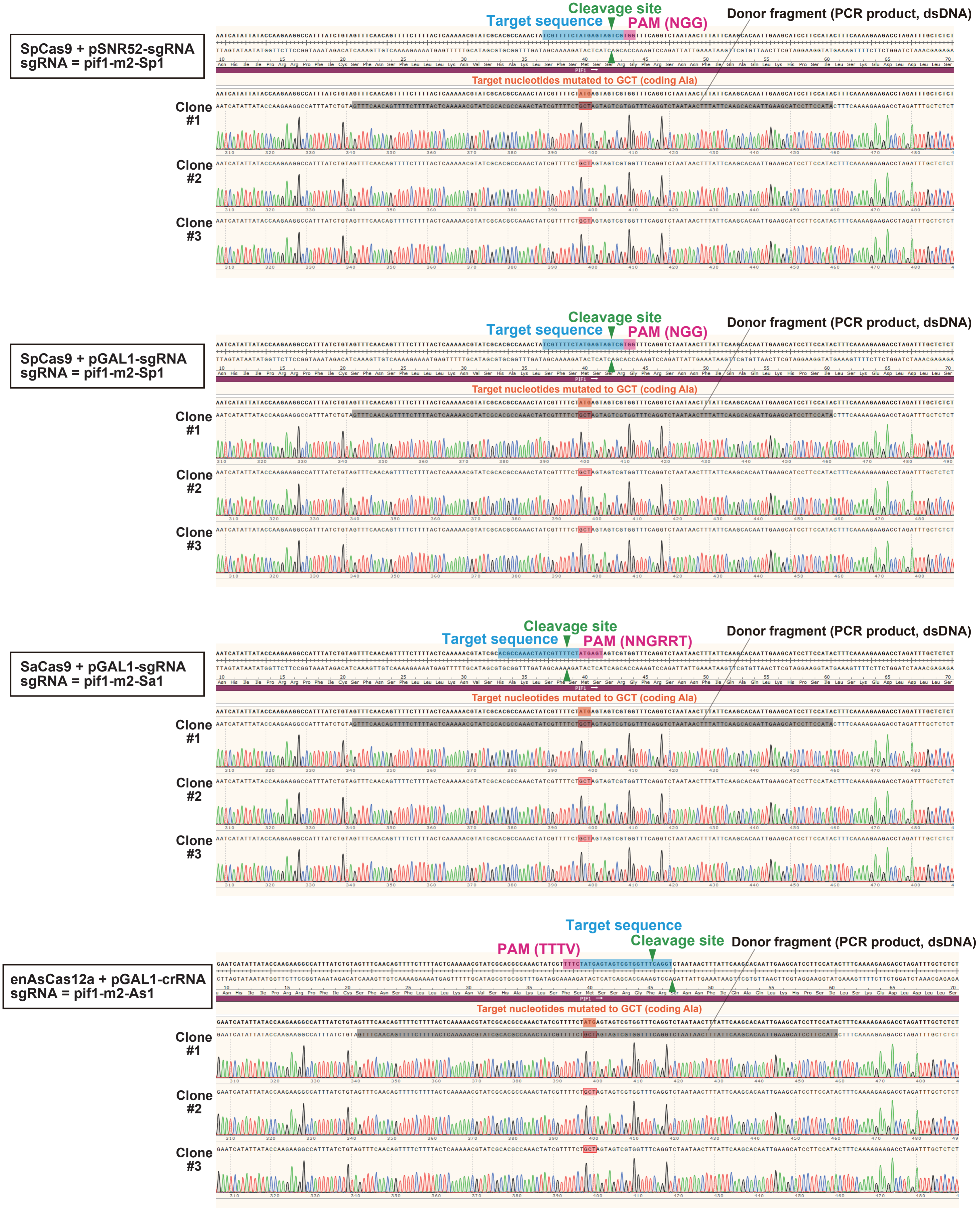

Figure S12. Nucleotide sequences of *pif1-m2* (M40A) clones generated by genome editing.
Nucleotide sequences are shown for the *pif1-m2* (M40A) clones generated by transformation of the individual genome-editing plasmids with the donor PCR fragment (Figure 5B). The wild-type *PIF1* sequence is shown at the top. The target sequence and the PAM are highlighted with blue and magenta, respectively. Green triangles indicate the expected Cas cleavage sites. Target nucleotides are colored in orange. The sequence of the donor fragment is colored in gray.

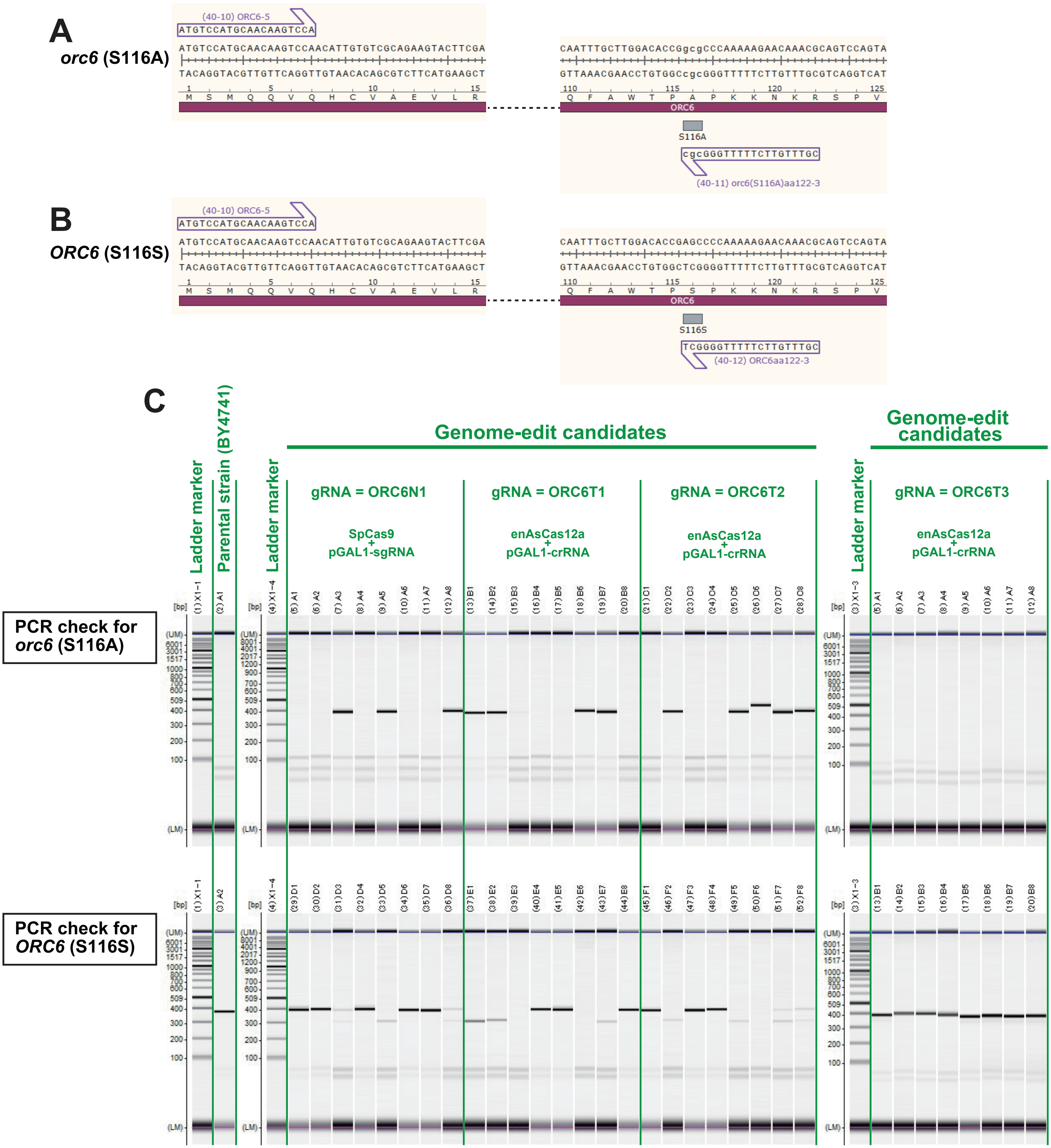

Figure S13. PCR examination of *orc6* (S116A) allele.
(A, B) Primers for PCR assay to distinguish the *orc6* (S116A) allele and the wild-type *ORC6* (S116S) allele. (C) An example of results of PCR assay to distinguish the *orc6* (S116A) allele and the wild-type *ORC6* (S116S) allele applied to candidate genome-edited clones. Top, PCR check for the orc6 (S116A) allele. Bottom, PCR check for the wild-type *ORC6* (S116S) allele. Each clone was examined using the two pairs of primers.

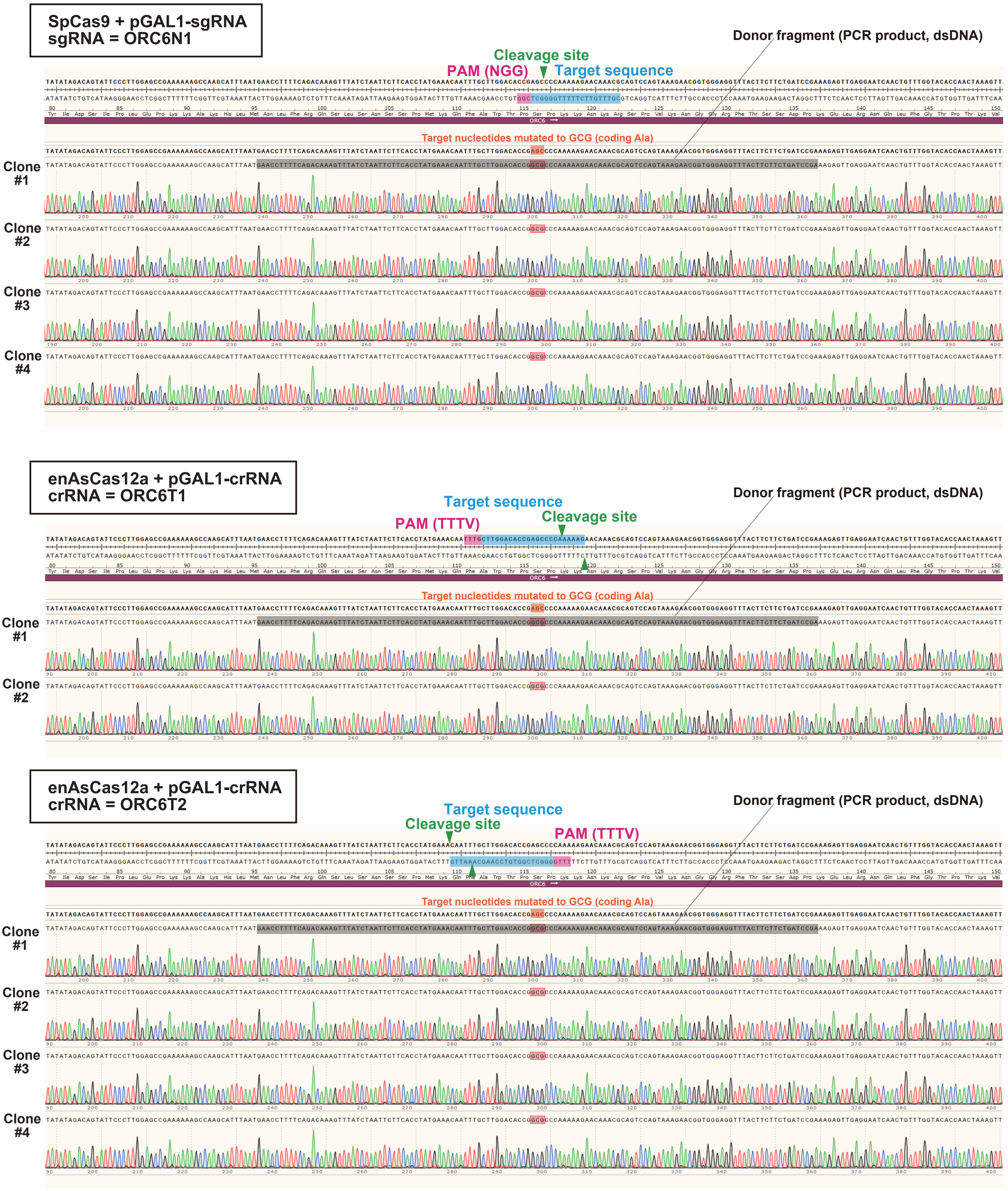

Figure S14. Nucleotide sequences of *orc6* (S116A) clones generated by genome editing.
Nucleotide sequences are shown for the *orc6* (S116A) clones generated by transformation of the individual genome-editing plasmids with the donor PCR fragment (Figure 5D). The wild-type *ORC6* sequence is shown at the top. The target sequence and the PAM are highlighted with blue and magenta, respectively. Green triangles indicate the expected Cas cleavage sites. Target nucleotides are colored in orange. The sequence of the donor fragment is colored in gray.

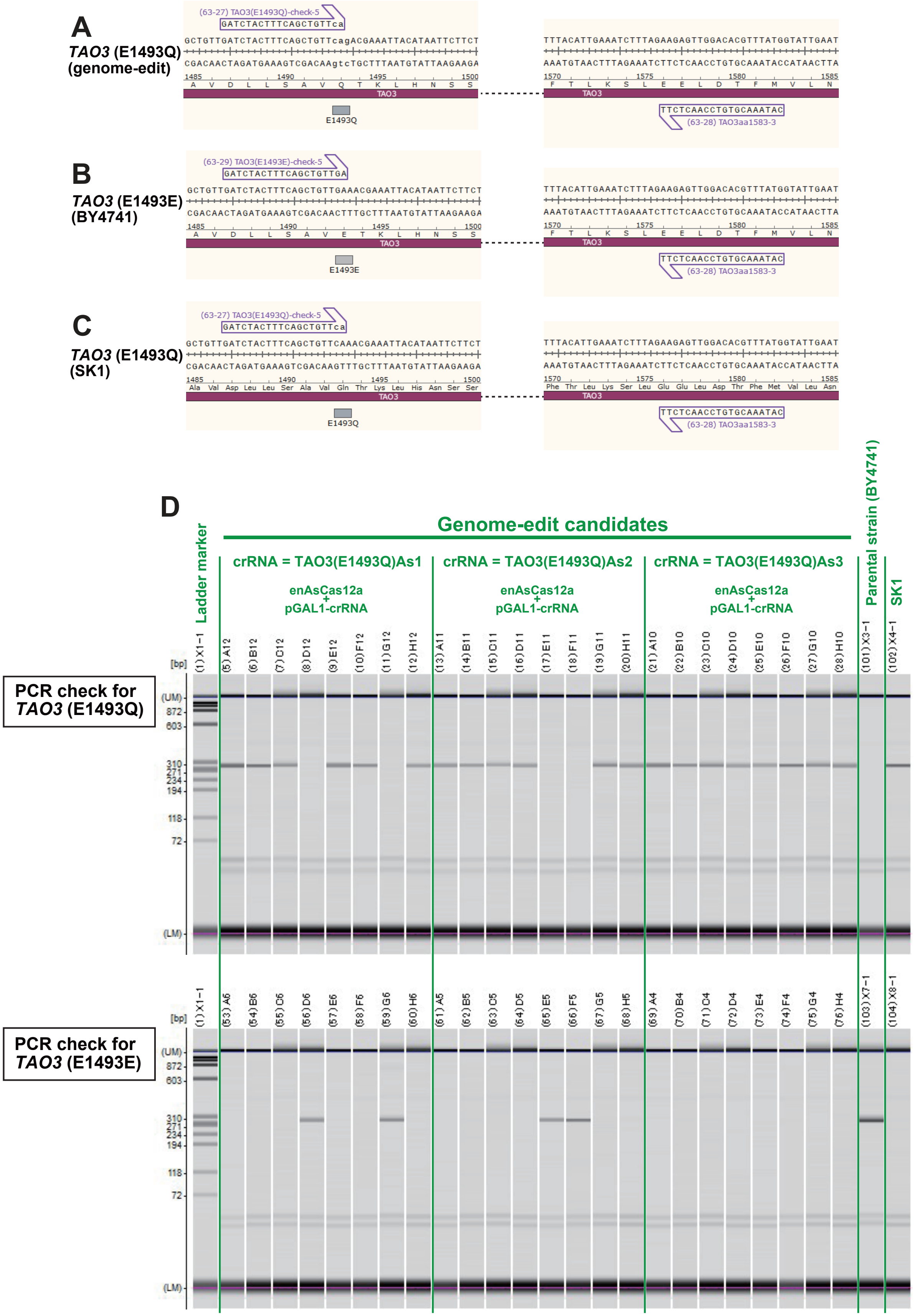

Figure S15. PCR examination of *TAO3* (E1493Q) allele.

(A, B, and C) Primers for PCR assay to distinguish the *TAO3* (E1493Q) alleles and the *TAO3* (E1493E, BY4741-type) allele. (D) An example of results of PCR assay to distinguish the *TAO3* (E1493Q) allele and the *TAO3* (E1493E, BY4741-type) allele applied to candidate genome-edited clones. Top, PCR check for the *TAO3* (E1493Q) allele. Bottom, PCR check for the *TAO3* (E1493E, BY4741-type) allele. Each candidate clone was examined using the two pairs of primers.

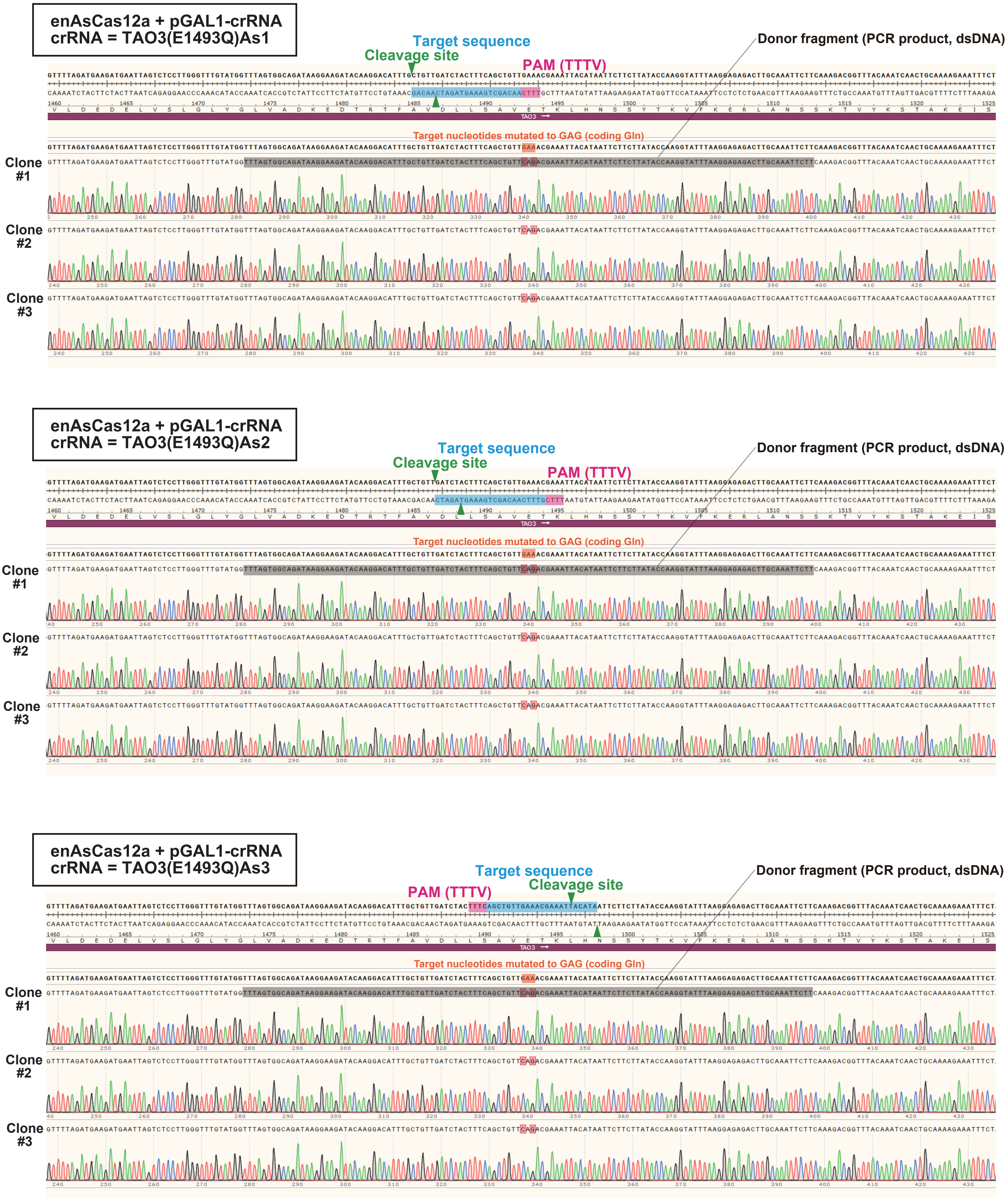

Figure S16. Nucleotide sequences of *TAO3* (E1493Q) clones generated by genome editing.
Nucleotide sequences are shown for the *TAO3* (E1493Q) clones generated by transformation of the individual genome-editing plasmids with the donor PCR fragment (Figure 5F). The BY4741-type *TAO3* (E1493E) sequence is shown at the top. The target sequence and the PAM are highlighted with blue and magenta, respectively. Green triangles indicate the expected enAsCas12a cleavage sites. Target nucleotides are colored in orange. The sequence of the donor fragment is colored in gray.
